# Chemotherapy-induced reactive myelopoiesis promotes expansion of immunosuppressive neutrophil-like monocytes in mice and humans

**DOI:** 10.1172/jci.insight.198360

**Published:** 2026-03-31

**Authors:** Huidong Shi, Zhi-Chun Ding, Ogacheko D. Okoko, Xin Wang, George Zhou, Yan Ye, Md Yeashin Gazi, Caitlin Brandle, Lirong Pei, Rafal Pacholczyk, Catherine C. Hedrick, Locke J. Bryan, Gang Zhou

**Affiliations:** 1 Georgia Cancer Center, Medical College of Georgia, and; 2 Immunology Center of Georgia, Augusta University, Augusta, Georgia, USA.

**Keywords:** Immunology, Oncology, Monocytes, Neutrophils

## Abstract

Cytotoxic chemotherapy primarily targets rapidly proliferating cancer cells but also depletes normal myeloid cells. The resulting cell loss triggers reactive myelopoiesis, a compensatory process in which hematopoietic stem and progenitor cells in the bone marrow (BM) regenerate myeloid lineages. We previously showed that the alkylating agent cyclophosphamide (CTX) induces myelopoiesis, leading to the expansion of immunosuppressive monocytes in mice. However, the molecular features and clinical relevance of these cells remain poorly understood. Here, we report the emergence of immunosuppressive monocytes in the peripheral blood of lymphoma patients receiving CTX-containing chemotherapy. To gain mechanistic insight into CTX-induced myelopoiesis, we performed single-cell RNA sequencing (scRNA-seq) and assay for transposase-accessible chromatin using sequencing (ATAC-seq) on BM monocytes from CTX-treated mice. These analyses revealed a heterogeneous monocyte population and demonstrated that CTX skews myelopoiesis toward the generation of neutrophil-like monocytes (NeuMo). Moreover, CTX-induced NeuMo cells, enriched within the CXCR4^+^CX3CR1^–^ monocyte subset, exhibited potent T cell–suppressive activity. Using the NeuMo gene signature, reanalysis of public scRNA-seq datasets identified a transcriptionally similar monocyte subset in chemotherapy-treated cancer patients. Collectively, our findings suggest that the expansion of NeuMo cells following chemotherapy represents a conserved immunoregulatory feedback mechanism with potential impact on tumor response to chemoimmunotherapy.

## Abstract 2

Chemotherapy promotes the expansion of neutrophil-like monocytes with T cell-suppressive activity, representing an immunoregulatory mechanism that may limit the therapeutic efficacy of cancer treatment.

## Introduction

Chemotherapy remains a primary treatment for cancer, effectively killing rapidly proliferating cancer cells and inducing remission. However, tumor recurrence due to acquired chemoresistance poses a significant clinical challenge. Increasing evidence suggests that the reactive responses of both cancer and noncancer cells to chemotherapy may counteract its efficacy (1–3). It has been shown that chemotherapy-induced alterations in the tumor microenvironment, including changes in immune cells, endothelial cells, and fibroblasts, can promote tumor survival, metastasis, and immune evasion (4–7). A better understanding of how chemotherapy reshapes noncancerous cell populations is essential for developing more effective treatment strategies that yield durable therapeutic outcomes.

Myeloid cells are a major component of the innate immune system, serving as the first line of defense against pathogens and tissue damage. The rapid depletion of myeloid cells following chemotherapy triggers a compensatory process known as reactive myelopoiesis, in which hematopoietic stem and progenitor cells (HSPCs) in the bone marrow (BM) regenerate granulocytes, monocytes, dendritic cells, and macrophages to restore immune homeostasis (8, 9). Myelopoiesis proceeds through a highly regulated, multistep differentiation hierarchy (10–12), in which HSPCs first give rise to common myeloid progenitors (CMPs). CMPs further differentiate into granulocyte-macrophage progenitors (GMPs) and monocyte–dendritic cell progenitors (MDPs). GMPs generate granulocytic precursors that mature into neutrophils, eosinophils, and basophils, while MDPs give rise to monocytes, dendritic cells, and macrophages.

Recent advances in single-cell RNA sequencing (scRNA-seq) have provided unprecedented insights into myeloid cell ontogeny and heterogeneity during myelopoiesis (11, 12). Studies in mice have demonstrated that monocytes can arise independently from both GMPs and MDPs, giving rise to transcriptionally distinct subsets, including neutrophil-like monocytes (NeuMo) and dendritic cell–like monocytes (DCMo) (10). NeuMo, derived from GMPs, exhibit a neutrophil-associated gene signature, including elastase (*Elane*), myeloperoxidase (*Mpo*), proteinase 3 (*Prtn3*), cathepsin G (*Ctsg*), and chitinase-like protein 3 (*Chil3*) (13–15). In contrast, DCMo, derived from MDPs, express genes related to MHC-II antigen presentation and dendritic cell markers (13, 16). Functionally, NeuMo and DCMo play distinct roles in different biological contexts (17). NeuMo expansion has been observed in systemic lipopolysaccharide exposure and brain injury models (13, 14, 18), while DCMo increase in aged mice and following unmethylated CpG DNA exposure (13, 16).

Despite these emerging insights, the relative abundance and function of NeuMo and DCMo in chemotherapy-induced myelopoiesis remain poorly understood. We previously reported that monocytes repopulating the BM and spleen after cyclophosphamide (CTX) chemotherapy in mice acquire a neutrophil precursor gene signature and immunosuppressive activity (19, 20). However, the relationship between these monocytes and either NeuMo or DCMo has yet to be defined. Furthermore, it remains unclear whether similar monocyte subsets arise in cancer patients undergoing chemotherapy. In this study, we report the emergence of immunosuppressive monocytes in the peripheral blood of a subset of lymphoma patients receiving CTX-containing standard-of-care (SOC) chemotherapy. To elucidate the impact of CTX on monocyte heterogeneity, we performed transcriptomic and chromatin accessibility profiling of mouse BM monocytes. Integrated analysis of scRNA-seq and assay for transposase-accessible chromatin using sequencing (ATAC-seq) data, combined with functional assays, reveals that chemotherapy-induced reactive myelopoiesis leads to the expansion of NeuMo cells with T cell–suppressive capacity. Furthermore, reanalysis of publicly available scRNA-seq datasets from cancer patient peripheral blood mononuclear cells (PBMCs) demonstrates transcriptional signatures consistent with NeuMo cells in individuals treated with SOC chemotherapy. Together, these findings provide, to our knowledge the first evidence that immunosuppressive NeuMo cells expand following chemotherapy in both preclinical models and cancer patients, offering new insights into how chemotherapy may reshape the myeloid cell compartment.

## Results

### Monocytes from lymphoma patients exhibit variable levels of immunosuppressive activity following chemotherapy.

**Figure 1:**
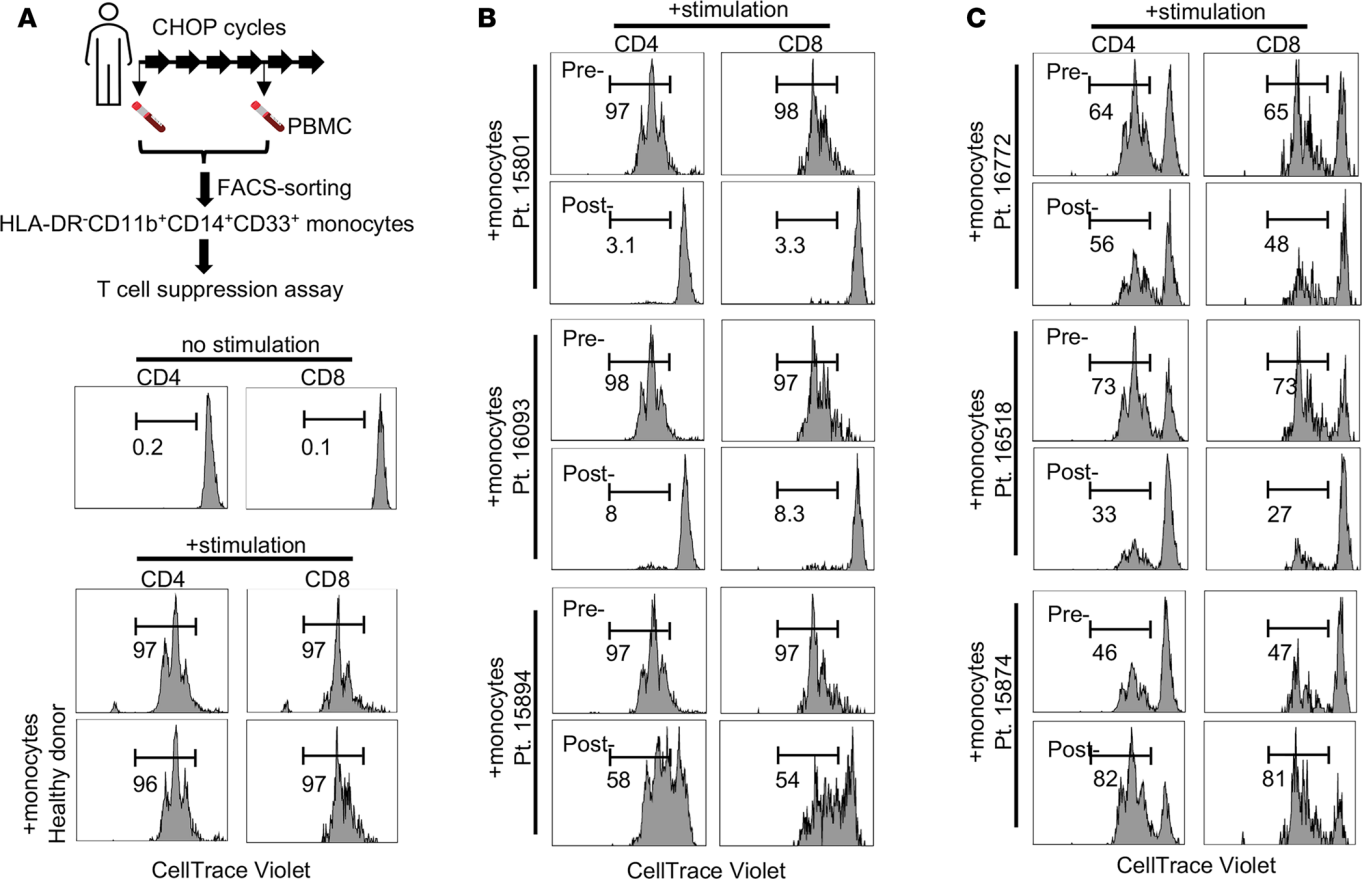
Emergence of immunosuppressive monocytes in the PBMCs of lymphoma patients following chemotherapy. (**A**) As depicted in the schema, patients received 6 cycles of standard-of-care chemotherapy. PBMC samples collected before chemotherapy and after 4 cycles of treatment were cryopreserved. HLA-DR^–^CD11b^+^CD14^+^CD33^+^ monocytes were FACS-sorted from pre- and post-chemotherapy PBMCs and used for in vitro T cell suppression assays. T cells isolated from a healthy donor were labeled with violet dye and used as responder cells. T cells were stimulated with Human T-Activator CD3/CD28 Dynabeads in the presence of pre- or post-chemotherapy monocytes. Cells were harvested 3 days later and evaluated for CD4^+^ and CD8^+^ T cell proliferation status by FACS. Violet dye histograms show T cell proliferation status under control cell culture conditions. The controls included T cells without stimulation (top row), T cells stimulated alone (middle row), and T cells stimulated in the presence of monocytes sorted from the PBMCs of a healthy donor (bottom row). (**B**) Patient samples showing that immunosuppressive monocytes were absent in pre-chemotherapy patient PBMCs but emerged in post-chemotherapy patient PBMCs. (**C**) Patient samples showing that immunosuppressive monocytes were present in pre-chemotherapy patient PBMCs. Post-chemotherapy monocytes from these patients showed either enhanced or reduced T cell suppression activities. Numbers in histograms represent percentage of divided T cells.

We previously reported that monocytes repopulating in mice after CTX chemotherapy acquire a neutrophil precursor gene signature and immunosuppressive activity (19, 20). CTX is a key component of several SOC chemotherapy regimens, including CHOP and EPOCH, which are widely used for the treatment of B cell lymphoma. However, whether chemotherapy-induced immunosuppressive monocytes arise in cancer patients receiving CTX-containing regimens remains unclear. To investigate this, we performed in vitro T cell suppression assays to evaluate the capacity of monocytes isolated from patient PBMCs to inhibit T cell activation. In this assay, responder T cells, containing both CD4^+^ and CD8^+^ T cells, were stimulated with anti-CD3 and anti-CD28 antibody–conjugated Dynabeads, either alone or in the presence of patient-derived monocytes. As shown in the schema in Figure 1A, deidentified, retrospective PBMC specimens collected from patients with B cell lymphoma before and after chemotherapy were used to sort HLA-DR^–^CD11b^+^CD33^+^ monocytes by FACS. To ensure consistency across experiments, the same responder T cells were isolated from the same healthy donor and used throughout. We analyzed monocytes collected from a total of 6 patients. As expected, CD4^+^ and CD8^+^ T cells proliferated robustly upon stimulation, with over 90% of cells undergoing cell division (Figure 1A). Addition of monocytes from a healthy donor had no effect on T cell proliferation (Figure 1A). Interestingly, monocytes from 3 patients exhibited de novo immunosuppressive activity after 4 cycles of chemotherapy, despite showing no suppressive capacity before treatment (Figure 1B). In the remaining 3 patients, monocytes already exhibited immunosuppressive activity before chemotherapy (Figure 1C, pretreatment samples). Notably, chemotherapy altered the suppressive capacity of these monocytes in different ways: in 2 patients (Pt. 16772 and Pt. 16518), immunosuppression was enhanced following treatment (Figure 1C, post-treatment samples), whereas in the third patient (Pt. 15874), monocyte-mediated suppression was reduced (Figure 1C, post-treatment sample). Our data suggest that the immunosuppressive status of monocytes in lymphoma patients is heterogeneous. Moreover, chemotherapy-induced myelopoiesis may contribute to the emergence of immunosuppressive monocytes in a subset of patients.

### scRNA-seq analysis of BM cells reveals the dynamic changes in myeloid cell composition during CTX-induced myelopoiesis in mice.

**Figure 2:**
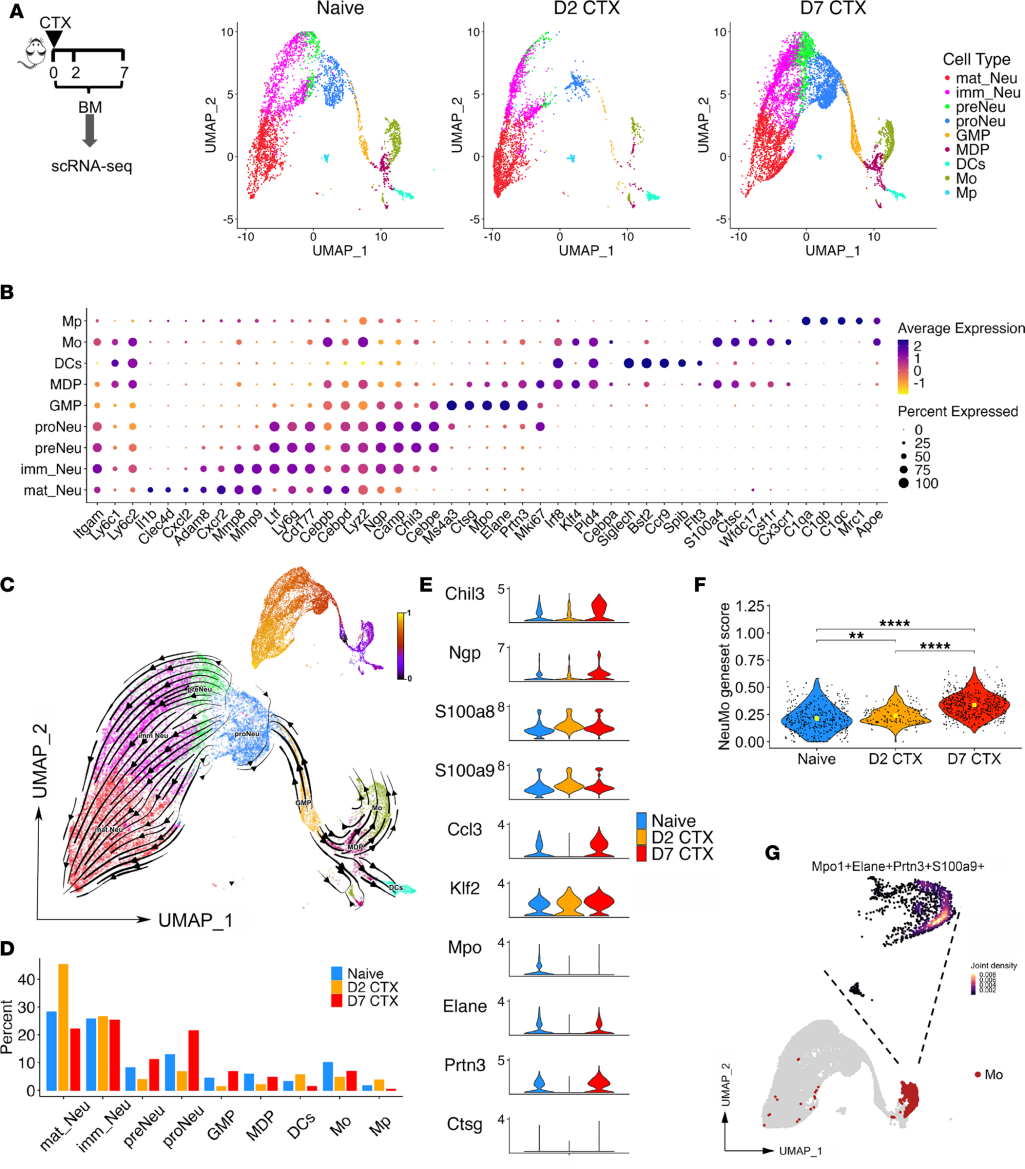
scRNA-seq analysis reveals the dynamic changes in immune composition in the BM following chemotherapy. (**A**) The procedures and timeline are depicted in the schema. Briefly, BM cells were collected from CTX-treated mice on days 2 and 7. BM cells from naive mice were used as controls. After removal of dead cells, the whole BM cells were subjected to 10x Genomics scRNA-seq analysis. UMAP plots show annotated myeloid cell populations based on gene expression profiles in BM samples from naive and day 2 and day 7 CTX-treated mice. (**B**) Dot plot of myeloid cell subtype–specific markers that distinguishes these cell populations. (**C**) RNA velocity and pseudotime analyses demonstrating lineage relationships and potential differentiation trajectories of myeloid cell subsets in response to chemotherapy. (**D**) Bar graph summarizing the proportions of each identified cell type across the 3 conditions. (**E**) Violin plots displaying expression levels of several known NeuMo marker genes in CD11b^+^ monocytes across naive and day 2 and day 7 samples. (**F**) Violin plot showing the NeuMo gene signature score, based on the gene set identified by Barman et al. (16). Pairwise comparisons were performed using 2-sided Wilcoxon’s rank-sum tests with Benjamini-Hochberg correction for multiple comparisons. **P < 0.01; ****P < 0.0001. (**G**) Nebulosa density plot illustrating the coexpression pattern of 4 NeuMo signature genes in monocyte subsets. High-density regions represent clusters of cells coexpressing these genes. mat_Neu, mature neutrophil; imm _Neu, immature neutrophil; preNeu, pre-neutrophil; proNeu, progenitor neutrophil; GMP, granulocyte-monocyte progenitor; MDP, monocyte–dendritic cell progenitor; DC, dendritic cell; Mo, monocyte; Mp, macrophage.

To better understand the dynamic changes in immune cell composition during CTX-induced myelopoiesis, we performed unbiased single-cell RNA sequencing (scRNA-seq) on BM cells from mice. As depicted in the schema in Figure 2A, BM samples were collected from mice 2 days (D2 CTX) and 7 days (D7 CTX) after CTX treatment, along with BM samples from untreated naive mice as controls. Across all conditions, we analyzed a total of 30,012 cells (10,890 naive, 9,765 D2 CTX, 9,357 D7 CTX), which were clustered into 20 distinct populations based on transcriptional profiles (Supplemental Figure 1, A–C; supplemental material available online with this article; https://doi.org/10.1172/jci.insight.198360DS1). Figure 2A illustrates the dynamic changes of major myeloid cell subtypes, which were identified using canonical marker genes shown in Figure 2B. To explore lineage relationships during myeloid reconstitution, we performed RNA velocity analysis, which infers cellular trajectories from spliced-to-unspliced transcript ratios. As shown in Figure 2C, GMPs were projected to differentiate into neutrophils through the progenitor neutrophil/pre-neutrophil/immature neutrophil pathway, while MDPs were predicted to give rise to monocytes and dendritic cells. These trajectory projections are consistent with the canonical hierarchy of myeloid differentiation, thereby validating our cell type annotations.

Assessment of BM cell composition over time revealed that CTX exposure led to the rapid depletion of multiple myeloid populations by day 2, including GMPs, progenitor neutrophils, pre-neutrophils, MDPs, and monocytes (Figure 2, A and D). By day 7, however, the frequencies of these myeloid subsets had rebounded and even exceeded pretreatment levels. The frequencies of T and B cells (with the exception of large pre–B cells) remained largely unaffected on day 2 but were substantially reduced by day 7 (Supplemental Figure 1B). To quantify these changes, total BM cell numbers were enumerated, and absolute numbers of T, B, and myeloid cells were calculated based on their frequencies determined by flow cytometry. Consistent with the scRNA-seq results, myeloid cell numbers, including both monocytes and neutrophils, declined 2 days after CTX but recovered and exceeded baseline levels by day 7, whereas B cell numbers remained robustly suppressed at this time point (Supplemental Figure 1, D and E). These results suggest that myelopoiesis precedes lymphopoiesis following CTX treatment, with monocytes and neutrophils rapidly repopulating the BM niche before the slower recovery of lymphoid cells.

Next, we investigated the presence and dynamics of neutrophil-like monocytes (NeuMo) in our dataset. Cells expressing a canonical NeuMo gene signature (*Mpo^+^Elane^+^Prtn3^+^Ctsg^+^*) were present in naive BM, decreased at day 2 after CTX, and re-emerged and expanded by day 7 (Figure 2, E and F). Notably, monocytes on day 7 also expressed elevated levels of *Chil3* and *Klf2*, along with other neutrophil-associated genes, including *Npg*, *S100a8*, and *S100a9* (Figure 2E). Cells coexpressing the NeuMo signature genes were identified within the monocyte cluster (Figure 2G) and displayed a temporal pattern consistent with depletion at day 2 and expansion at day 7 (Figure 2, E and F). Together, these findings demonstrate that CTX-induced myelopoiesis perturbs monocyte homeostasis and promotes the expansion of a transcriptionally distinct NeuMo-like population within the BM. This highlights a previously underappreciated aspect of chemotherapy-driven immune remodeling.

### scRNA-seq analysis of classical monocytes in the BM identifies NeuMo and DCMo cell subsets.

**Figure 3:**
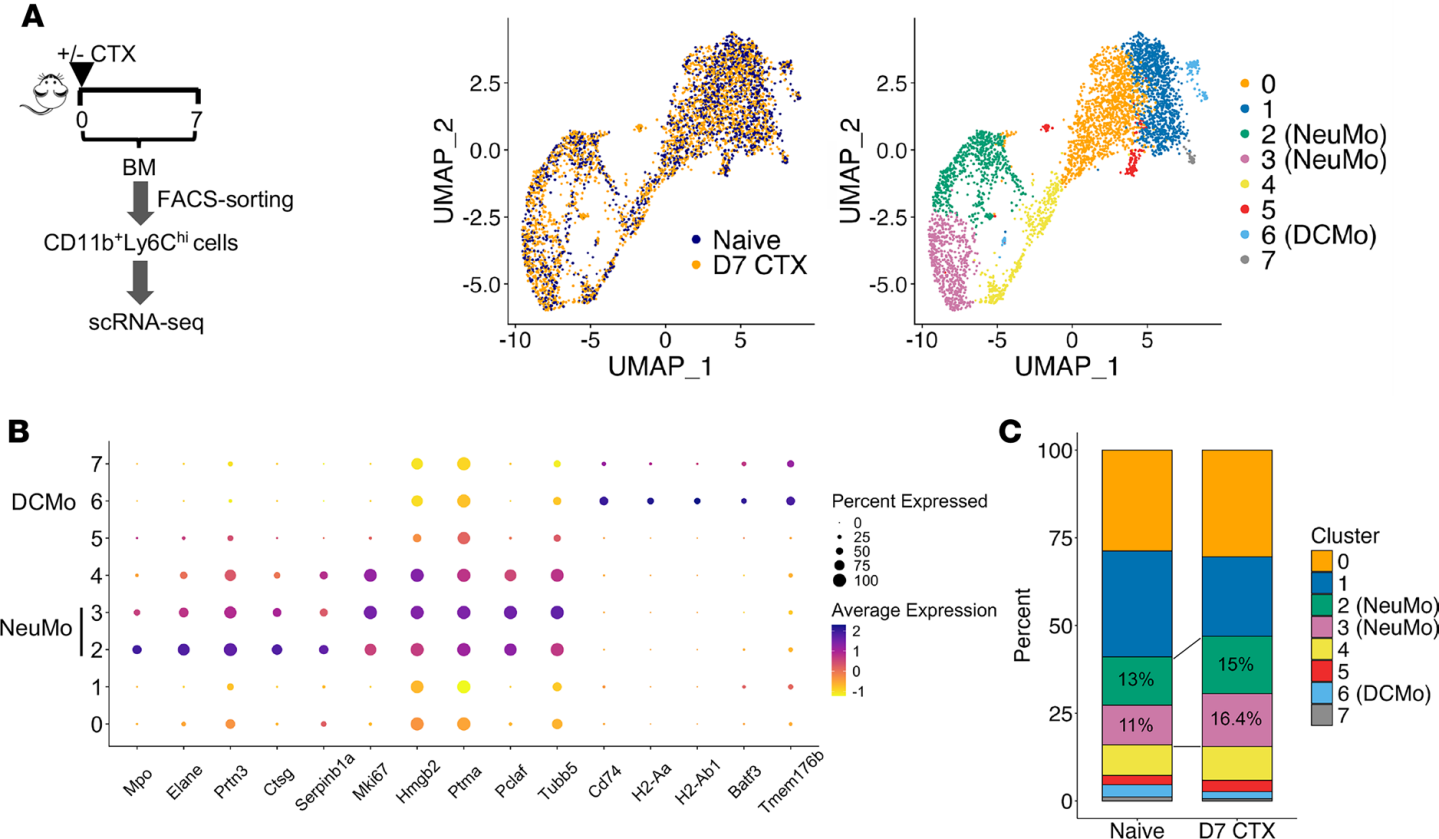
scRNA-seq analysis of pre-enriched monocytes (CD11b^+^Ly6C^hi^) identifies distinct cell subsets based on gene expression profiles. (**A**) The schema depicts the procedures of sample collection. Briefly, BM cells were collected from CTX-treated mice on day 7. BM cells from naive mice were used as controls. CD11b+Ly6Chi classical monocytes were isolated from BM by cell sorter. Sorted cells were subjected to scRNA-seq. UMAP plots show cell distribution by sample origin (left) and cell cluster/subset identity (right). (**B**) Dot plot highlighting representative marker genes for NeuMo (clusters 2 and 3) and DCMo (cluster 6). Dot color indicates the average expression level of each gene, while dot size reflects the proportion of cells within each cluster expressing that gene. (**C**) Bar graph summarizing the percentage distribution of each cluster across the analyzed samples. The proportions of clusters 2 and 3 (NeuMo populations) are indicated.

Recent studies have revealed heterogeneity within classical monocytes (CD11b^+^Ly6C^hi^), identifying at least 2 distinct subsets, i.e., NeuMo and DCMo, based on transcriptomic profiling (13, 16–18, 21). While our scRNA-seq analysis of whole BM provided valuable insights into CTX-induced changes in the myeloid compartment (Figure 2), the limited number of monocytes in those datasets restricted our ability to characterize monocyte heterogeneity in detail. To achieve higher resolution, we performed scRNA-seq on pre-enriched CD11b^+^Ly6C^hi^ classical monocytes isolated from the BM of naive mice and mice 7 days after CTX treatment (schema in Figure 3A). A total of 15,135 monocytes were analyzed (6,512 from naive mice and 8,623 from D7 CTX mice). Using previously published NeuMo and DCMo gene signatures (12), we identified 2 monocyte clusters (clusters 2 and 3) that expressed high levels of NeuMo-associated genes, including *Mpo*, *Elane*, *Prtn3*, and *Ctsg* (Figure 3, A and B). Interestingly, cluster 3 NeuMo cells showed elevated expression of proliferation-related genes such as *Mki67*, *Hmgb2*, *Ptma*, *Pclaf*, and *Tubb5*, suggesting a more proliferative phenotype than cluster 2 NeuMo cells. In addition, we identified a small subset of cells (cluster 6) expressing the DCMo signature genes, including *Cd74*, *H2-Aa*, *H2-Ab1*, and *Batf3*. As shown in Figure 3C, both NeuMo and DCMo subsets were present in naive BM; however, the proportion of NeuMo cells (clusters 2 and 3 combined) increased by day 7 after CTX treatment, while DCMo cells remained a minor subset. These findings provide evidence that chemotherapy-induced myelopoiesis leads to preferential expansion of monocytes with a NeuMo transcriptional identity.

### CTX-induced epigenetic changes in monocytes correlate with the expansion of NeuMo cells.

**Figure 4:**
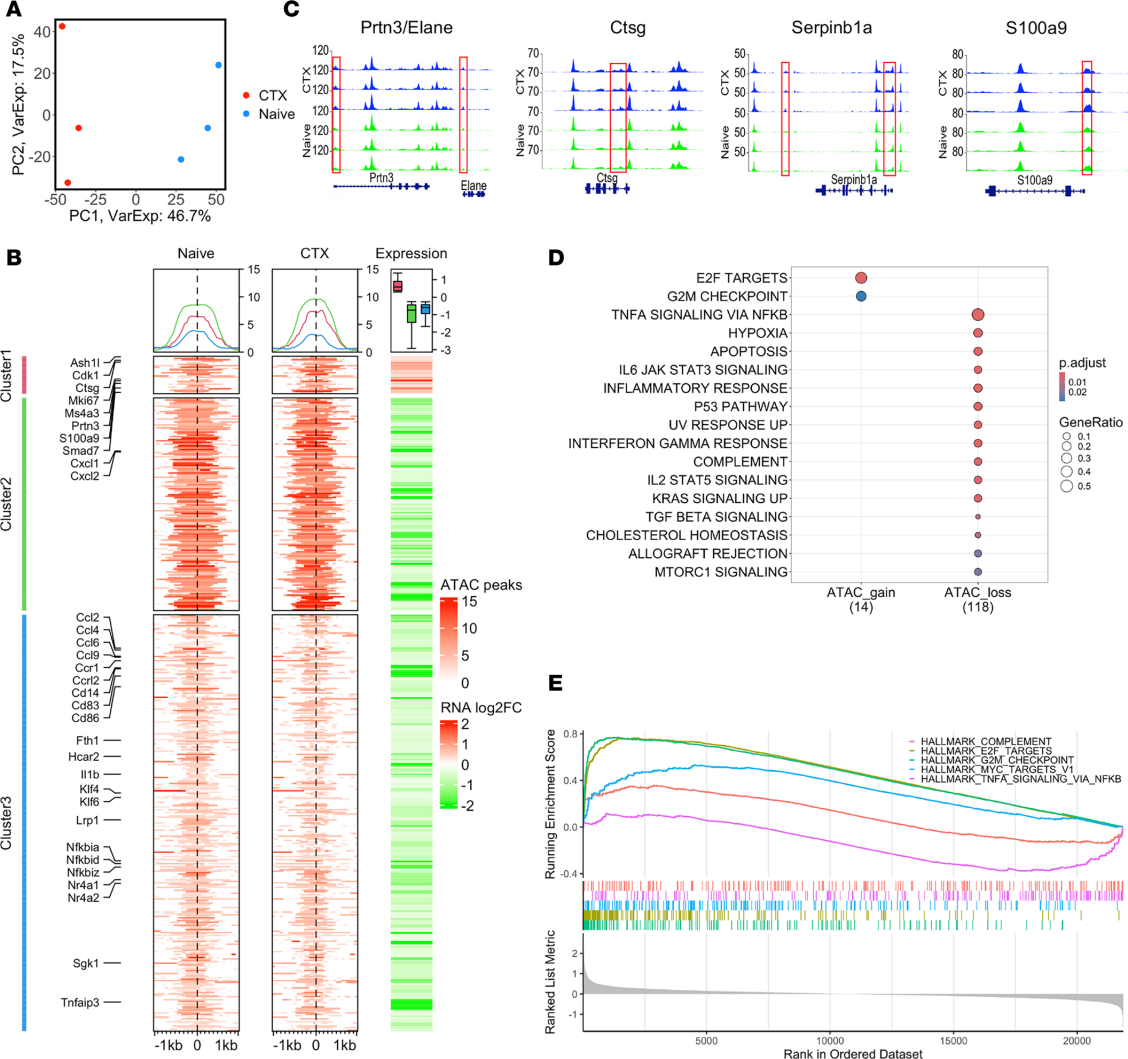
ATAC-seq analysis reveals chemotherapy-induced epigenetic changes in monocytes. Highly enriched monocytes (CD11b^+^Ly6C^hi^) from the BM of naive and CTX-treated mice were subjected to ATAC-seq. (**A**) Principal component analysis (PCA) plot of ATAC-seq data. The colors indicate the sample groups. (**B**) Heatmaps identifying differential chromatin accessibility (±2 kb windows centered at the summit of ATAC-seq peaks) between treated and untreated monocytes (left 2 columns) and gene expression fold changes from pseudo-bulk analysis of scRNA-seq data between these same 2 samples (fold change [FC] on the right). (**C**) BigWig plots of ATAC-seq data comparing chromatin accessibility in monocytes from control and CTX-treated mice. The representative loci (*Prtn3*/*Elane*, *Ctsg*, *Serpinb1a*, *S100a9*) that exhibit increased chromatin accessibility following CTX treatments are shown. (**D**) Dot plot of pathway enrichment analysis based on the rank of genes whose expression positively correlates with differential chromatin accessibility peaks between control and CTX-treated monocytes. Dot size represents the number of genes that overlap with pathway gene sets, while color represents adjusted *P* values. (**E**) Gene set enrichment analysis (GSEA) using all differentially expressed genes in BM monocytes from control and CTX-treated mice shows enriched pathways associated with E2F targets, cell cycle, and MYC target genes, with upregulation of these genes. In contrast, NF-κB–mediated TNF-α signaling–related genes are downregulated.

Using a lineage tracing approach, a recent study revealed that monocyte fate is epigenetically predetermined at the progenitor stage, implying distinct epigenetic profiles among monocyte subtypes (22). We performed ATAC-seq on sorted CD11b^+^Ly6C^hi^ BM monocytes from naive and CTX-treated mice to assess chromatin accessibility as a measure of epigenetic remodeling. Principal component analysis (PCA) revealed distinct chromatin accessibility profiles between monocytes from naive and CTX-treated mice (Figure 4A). Integrative analysis of ATAC-seq and scRNA-seq data of the sorted CD11b^+^Ly6C^hi^ monocytes from the BM of naive and CTX-treated mice identified 618 differential ATAC peaks between the 2 groups that were positively correlated with the expression of their nearest genes. Notably, only about 5% of these peaks (cluster 1) were associated with both increased chromatin accessibility and elevated transcription, while the majority (clusters 2 and 3) showed reduced chromatin accessibility and decreased gene expression (Figure 4B). Consistent with our scRNA-seq findings of NeuMo expansion in CTX-treated mice (Figure 3), several NeuMo-associated genes, including *Prtn3*, *Ctsg*, *Serpinb1a*, and *S100a9* (13), exhibited increased chromatin accessibility in monocytes following CTX treatment (Figure 4C). Additionally, proliferation-related genes such as *Mki67* and *Ezh2* were also among those with elevated chromatin accessibility and expression (Figure 4B), supporting the expansion of a proliferative NeuMo subset. Conversely, several inflammatory chemokines and chemokine receptors, such as *Ccl4*, *Ccl6*, *Ccl9*, *Ccr1*, and *Ccrl2*, were among those downregulated genes associated with loss of chromatin accessibility. Pathway enrichment analysis revealed that genes with increased accessibility were predominantly involved in cell cycle–related processes. In contrast, genes with reduced accessibility were enriched in inflammatory response, interferon signaling, and TNF-α signaling pathways (Figure 4D). These chromatin accessibility changes were further corroborated by gene set enrichment analysis (GSEA) (Figure 4E). Our results suggest that CTX remodels chromatin accessibility in gene loci linked to NeuMo differentiation and expansion within BM monocytes.

### NeuMo cells in the BM of CTX-treated mice exhibit immunosuppressive activity.

**Figure 5:**
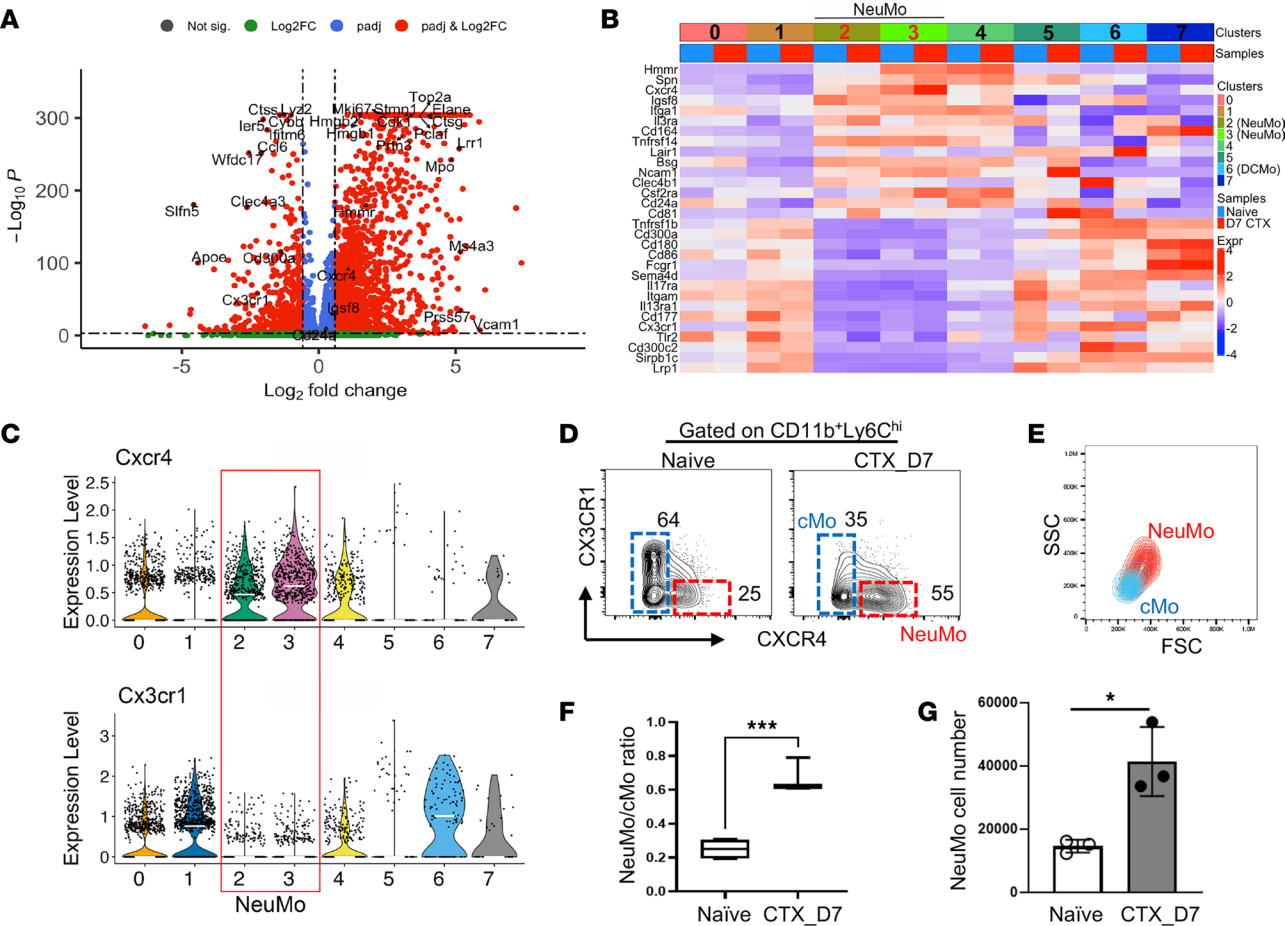
Identification of cell surface markers suitable for enrichment of CTX-induced NeuMo cells. (**A**) Volcano plot showing genes differentially expressed in NeuMo cells compared with other classified monocytes. Differential gene expression analysis was performed using the MAST test implemented in Seurat, and *P* values were adjusted for multiple testing using the Bonferroni method. (**B**) Heatmap of top 30 differentially expressed surface marker genes showing their expression levels across different monocyte clusters identified in Figure 3. (**C**) Violin plots illustrating the expression levels of Cxcr4 and Cx3cr1 used to define NeuMo cells. (**D**) The combination of CXCR4 and CX3CR1 can be used to distinguish NeuMo from other classical monocytes (cMo) within the BM monocyte population. The numbers in dot plots demarcate the percentages of NeuMo and other monocytes in BM-derived monocytes. (**E**) NeuMo cells show increased size (forward scatter [FSC]) and granularity (side scatter [SSC]) compared with other monocytes. (**F**) The ratio of NeuMo over other monocytes increases in the BM after CTX treatment. The ratio is calculated based on the data shown in **D** and shown as mean ± SEM with 3 mice in each group. (**G**) Absolute numbers of NeuMo-like cells in the BM were calculated based on cell percentages determined by flow cytometry and enumerated BM cells. The results are summarized in the bar graph (mean ± SEM). Data shown are representative of 2 independent experiments with similar results. Statistical analysis was performed using 2-tailed unpaired *t* test with Welch’s correction. *P < 0.05; ***P < 0.001.

To enable the prospective enrichment of NeuMo cells for downstream functional studies, we interrogated our monocyte scRNA-seq dataset to identify genes differentially expressed in NeuMo cells compared with other classical monocytes (Figure 5A). From these differentially expressed genes, we prioritized those encoding surface proteins and selected a panel of candidate markers based on their expression patterns across monocyte clusters (Figure 5B). Among them, *Cxcr4* and *Cx3cr1* were chosen as potential markers owing to their largely non-overlapping expression between NeuMo and other classical monocyte populations (Figure 5C), as well as their previously established roles in monocyte subset differentiation and trafficking (23, 24). Flow cytometry analysis using antibodies against CD11b, Ly6C, CXCR4, and CX3CR1 can effectively distinguish putative NeuMo cells (CD11b^+^Ly6C^hi^CXCR4^+^CX3CR1^lo^) from other classical monocytes (Figure 5D). Further phenotypic analysis revealed that the putative NeuMo population exhibited increased forward scatter (FSC) and side scatter (SSC) values (Figure 5E), indicative of larger cell size and greater granularity, features consistent with previously reported characteristics of NeuMo cells (13). Notably, the NeuMo/classical monocyte ratio was markedly elevated in the BM of CTX-treated mice (Figure 5F). Quantification of NeuMo cells based on CD11b^+^Ly6C^hi^CXCR4^+^CX3CR1^lo^ markers confirmed their expansion in the BM of CTX-treated mice (Figure 5G). Correspondingly, an increased frequency of CXCR4^+^CX3CR1^lo^ monocytes was also observed in the peripheral blood of CTX-treated mice (Supplemental Figure 2), suggesting systemic dissemination of NeuMo cells. These results establish a flow cytometry–based strategy for isolating live NeuMo cells from BM, facilitating further investigation into their functional roles in chemotherapy-induced immune modulation.

**Figure 6:**
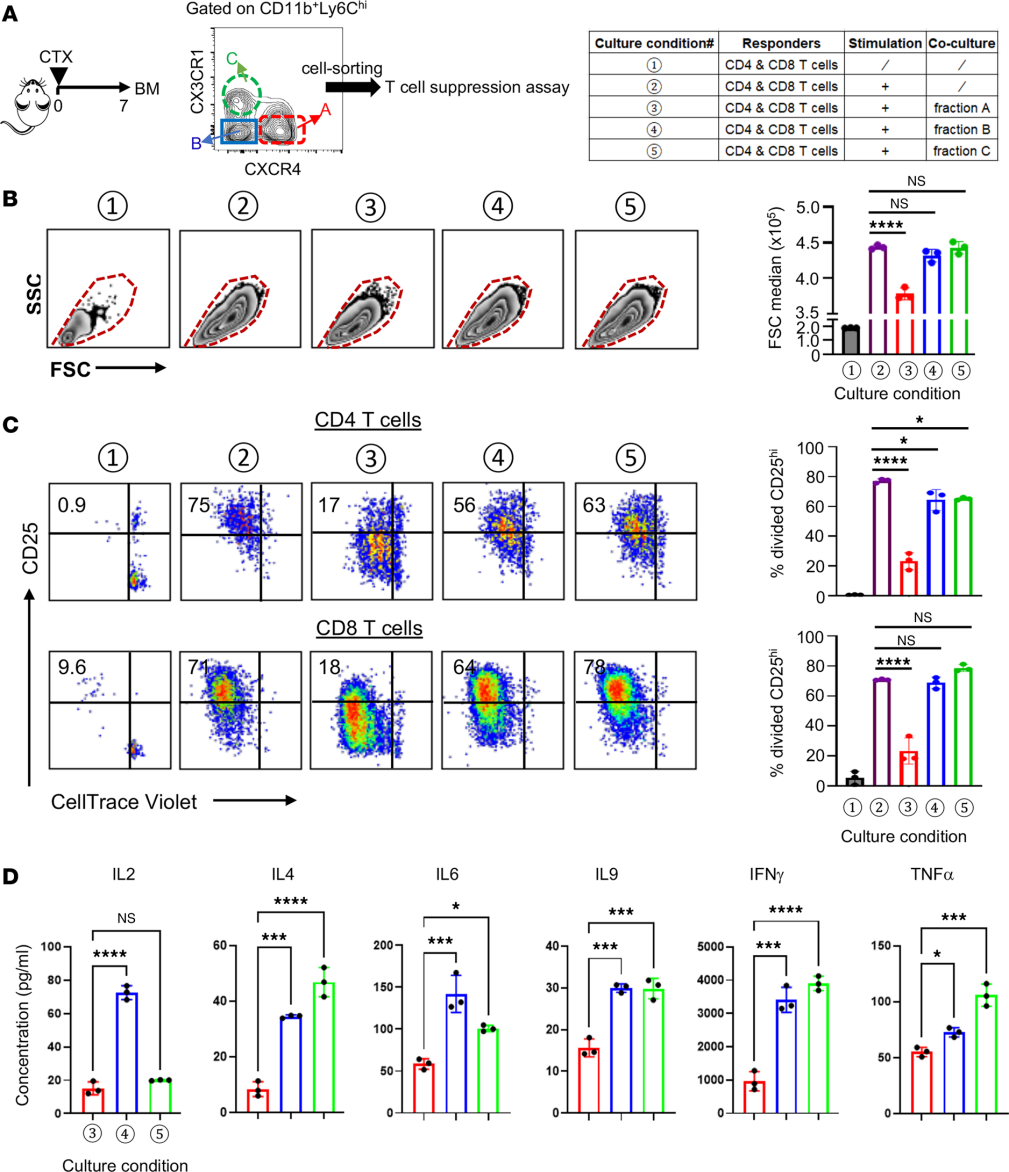
CTX-induced NeuMo cells possess the ability to suppress T cell activation. (**A**) Isolation of CTX-induced NeuMo cells for T cell suppression assays. The schema depicts the experimental procedures and timeline. Seven days after CTX treatment, BM monocytes (CD11b^+^Ly6C^hi^) were subjected to FACS sorting to isolate the indicated cell fractions based on CXCR4 and CX3CR1 expression patterns. The sorted monocytes were used for in vitro T cell suppression assay. The cell culture setup conditions are outlined in the table on the right. Three days after culture, cells were harvested for analysis. (**B**) Representative dot plots showing the size (FSC) and granularity (SSC) of cultured cells. Cells within the dashed red lines were primarily T cells. The median FSC values of the gated cells are summarized in the bar graph at right, shown as mean ± SEM of triplicate samples. (**C**) Cell division and activation status of the responder T cells. Cells were stained with antibodies against CD4, CD8, and CD25. Representative dot plots are gated on CD4^+^ (top panel) and CD8^+^ (bottom panel) T cells to show cell division status (violet dye dilution) and CD25 expression level. Numbers in dot plots represent percentage of fully activated T cells (divided CD25^hi^) under the specified culture condition. The results are summarized in the bar graphs at right, shown as mean ± SEM of triplicate samples. (**D**) Quantification of cytokines in cell culture. Supernatants from the indicated cell culture conditions were collected at time of cell harvest and subjected to LEGENDplex Multiplex Cytokine Assay to measure the concentrations of the indicated cytokines. Results are shown as mean ± SEM of triplicate samples. Data shown are representative of 2 independent experiments with similar results. Statistical analysis was performed using 1-way ANOVA with Tukey’s multiple-comparison test. *P < 0.05; ***P < 0.001; ****P < 0.0001.

Given previous reports demonstrating the immunoregulatory properties of NeuMo cells (14, 25), we investigated whether the NeuMo cells emerging in the BM following chemotherapy exhibit similar immunosuppressive functions. To this end, CD11b^+^Ly6C^hi^ classical monocytes from the BM of CTX-treated mice were FACS-sorted into 3 fractions based on their CXCR4 and CX3CR1 expression profiles, with fraction A (CXCR4^+^CX3CR1^–^) being enriched for NeuMo cells (Figure 6A). To assess their immunomodulatory capacity, we cocultured sorted monocyte fractions with splenocytes from naive mice, which contained both CD4^+^ and CD8^+^ T cells, in the presence of anti-CD3 and anti-CD28 antibodies to stimulate T cell activation (Figure 6A, table). As expected, T cells activated in the absence of monocytes showed increased FSC, indicative of activation-induced cell enlargement. Notably, coculture with fraction A monocytes substantially reduced T cell FSC, suggesting impaired activation, whereas monocytes from other fractions had no such effect (Figure 6B). Further phenotypic analysis revealed that, under stimulation, both CD4^+^ and CD8^+^ T cells underwent robust proliferation and upregulated CD25 (IL-2 receptor α), a hallmark of productive T cell activation (Figure 6C, condition 2). However, in the presence of fraction A monocytes, proliferating T cells displayed a dramatic reduction in CD25 expression (Figure 6C, condition 3), indicating an abortive activation phenotype. In contrast, T cells cocultured with monocytes from other fractions retained CD25 expression in CD8^+^ T cells, and only a modest reduction in CD25 was observed in CD4^+^ T cells (Figure 6C, conditions 4 and 5). Moreover, cocultures containing fraction A monocytes (condition 3) produced substantially lower levels of cytokines, including IL-2, IL-4, IL-6, IL-9, IFN-γ, and TNF-α, compared with cultures containing other monocyte subsets (Figure 6D). Comparison of markers associated with antigen presentation revealed lower levels of MHC-II and CD80 in NeuMo cells compared with other classical monocytes (Supplemental Figure 3). Interestingly, the NeuMo cells (CD11b^+^Ly6C^hi^CXCR4^+^CX3CR1^–^) isolated from naive BM only slightly reduced T cell proliferation and had minimal effect on CD25 upregulation (Supplemental Figure 4A). Their suppressive potency was substantially weaker than that of NeuMo cells derived from CTX-treated mice (Supplemental Figure 4B). Together, these results demonstrate that chemotherapy induces the expansion of NeuMo cells, enriched within the CXCR4^+^CX3CR1^–^ monocyte subset in the BM, where they acquire enhanced immunosuppressive activity capable of inhibiting T cell activation and cytokine production.

### Identification of NeuMo cells in patient PBMCs following chemotherapy.

**Figure 7:**
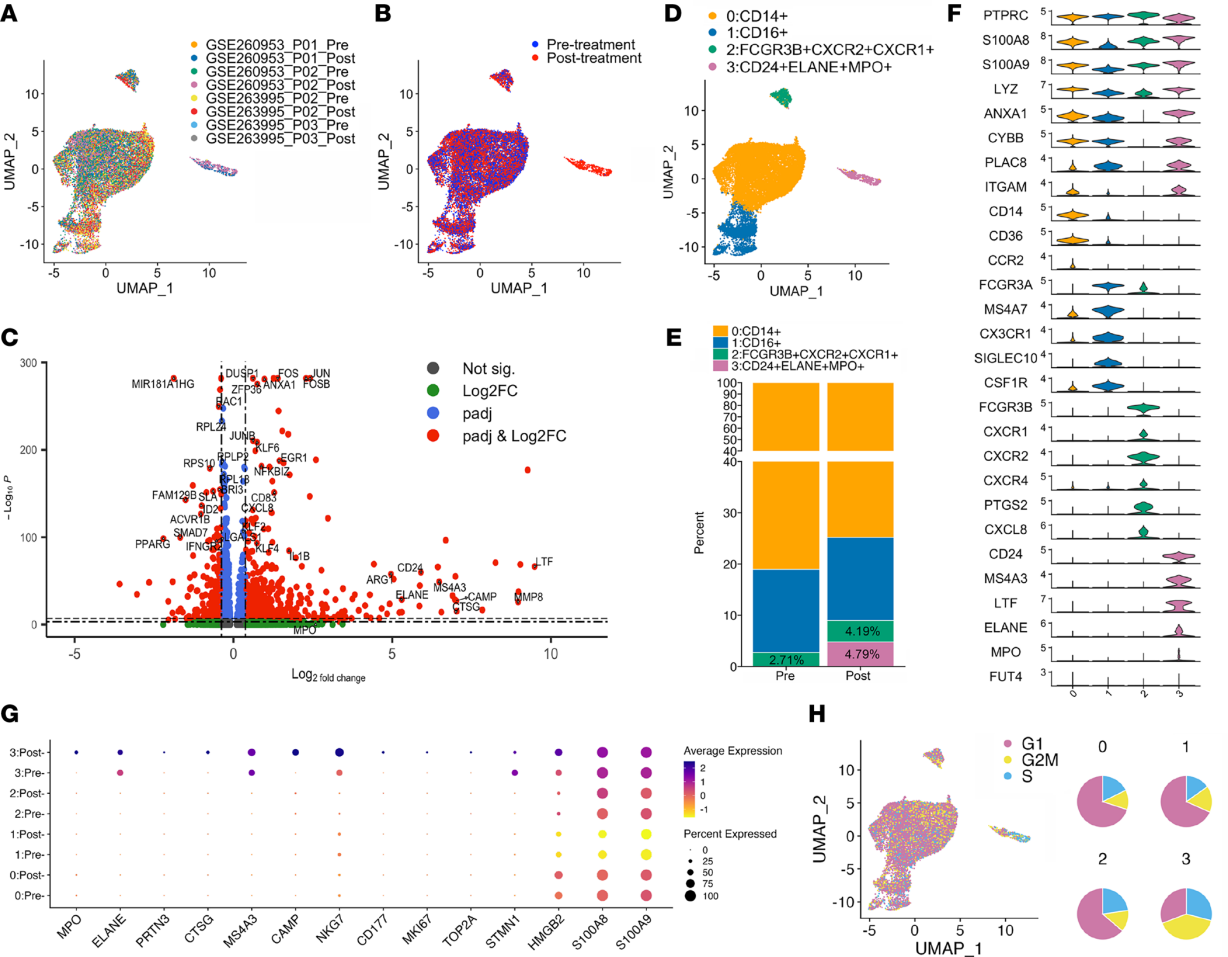
Reanalysis of publicly available scRNA-seq datasets identifies NeuMo-like cells emerging after chemotherapy in the PBMCs of cancer patients. (**A**) UMAP plot of 18,656 monocytes extracted from 2 publicly available scRNA-seq datasets of cancer patients, with cells colored by sample origin. (**B**) UMAP plot showing the same cells colored by chemotherapy treatment status. (**C**) Volcano plot displaying differentially expressed genes between pre- and post-chemotherapy monocytes. Differential gene expression analysis was performed using the MAST test implemented in Seurat, and *P* values were adjusted for multiple testing using the Bonferroni method. (**D**) UMAP clustering identifies 4 distinct monocyte subtypes, including classical (CD14^+^) and non-classical (CD16^+^) monocytes and 2 smaller clusters impacted by chemotherapy treatment. (**E**) Bar plot representing the proportions of each monocyte cluster in pre- and post-chemotherapy samples. (**F**) Violin plots showing expression of marker genes used to define the clusters in **D**. (**G**) Dot plot showing the expression of selected NeuMo-associated genes identified from mouse scRNA-seq datasets across the human monocyte subclusters, highlighting increased expression in cluster 3 cells following chemotherapy. (**H**) UMAP plot depicting the inferred cell cycle phase (G_1_, S, or G_2_/M) of each cell, with accompanying pie charts summarizing the distribution of cell cycle phases within each of the 4 monocyte clusters.

To validate our findings in a broader patient cohort, we analyzed 2 publicly available scRNA-seq datasets containing PBMCs collected from patients before and after chemotherapy. Specifically, we included 4 patients with paired pre- and post-treatment PBMC samples: 2 triple-negative breast cancer patients treated with CTX and doxorubicin (Gene Expression Omnibus [GEO] GSE263995) and 2 tongue squamous cell carcinoma patients treated with docetaxel, cisplatin, and 5-fluorouracil (GSE260953). Our objective was to determine whether NeuMo cells emerge following chemotherapy across distinct cancer types and therapeutic regimens. scRNA-seq data were downloaded from GEO and reference-mapped against publicly available PBMC scRNA-seq datasets from healthy donors (Supplemental Figure 5). From PBMC populations, we extracted 18,665 high-quality monocytes for further analysis (Figure 7A). Integration of the 8 scRNA-seq datasets revealed a unique monocyte population that emerged only after chemotherapy (Figure 7B). Pseudo-bulk differential expression analysis revealed upregulation of NeuMo gene signatures, including *MMP8*, *CAMP*, *ELANE*, and *CTSG*, in post-treatment monocytes (Figure 7C). Uniform manifold approximation and projection (UMAP) revealed classical CD14^+^ monocytes (cluster 0), non-classical CD16^+^ monocytes (cluster 1), and 2 minor populations expressing neutrophil lineage markers: one subpopulation characterized by *CD16B* and multiple CXCR receptors (cluster 2) and another characterized by NeuMo gene signatures (cluster 3) (Figure 7D). Notably, clusters 2 and 3 expanded following chemotherapy, increasing from 2.75% to 4.19% and from 0.2% to 4.78%, respectively, while classical (cluster 0) and non-classical (cluster 1) monocytes showed a modest decline (Figure 7E). Cluster 3 cells expressed *CD24* but lacked *CX3CR1*, mirroring the murine NeuMo profile. However, unlike their murine counterparts, these cells did not express *CXCR1*, *CXCR2*, or *CXCR4* (Figure 7F). In contrast, cluster 2 cells, which also expanded after chemotherapy, expressed these CXCR genes but also lacked *CX3CR1*. To further dissect the transcriptional differences between the cell clusters, we temporally broke down each cluster (pre- versus post-chemotherapy) and examined the expression levels of a selected panel of NeuMo-associated genes identified from murine scRNA-seq datasets (Figure 7G). Cluster 3 cells from post-chemotherapy samples exhibited elevated expression of genes associated with granulocyte-monocyte progenitors (*MPO*, *ELANE*, *CTSG*, *PRTN3*), proliferation (*MKI67*, *TOP2A*, *STMN1*), and immature neutrophil markers (*CD177*, *CAMP*, *NKG7*). Cell cycle analysis revealed an increased proportion of cluster 3 cells in the G_2_/M and S phases, indicating active proliferation (Figure 7H). Collectively, these findings demonstrate that cluster 3 monocytes from human PBMCs after chemotherapy share key transcriptional features with the NeuMo population identified in our murine model, supporting the emergence of a conserved, treatment-induced immunomodulatory monocyte subset.

## Discussion

Chemotherapeutic agents used to eliminate rapidly proliferating cancer cells also inevitably affect normal cells, including myeloid cells and other immune populations. Myeloid cell regeneration following chemotherapy, known as reactive myelopoiesis, occurs in the BM to replenish monocytes and neutrophils depleted by treatment. Our study provides a detailed analysis of the dynamic changes within the myeloid compartment during this regeneration process and identifies a distinct subset of neutrophil-like monocytes, also called NeuMo, that expands following chemotherapy.

Beyond the classical, intermediate, and non-classical monocyte subsets traditionally defined by surface markers, scRNA-seq analyses have revealed additional functional and developmental diversity within classical monocytes. Notably, NeuMo and DCMo represent 2 transcriptionally distinct monocyte subsets thought to originate from GMPs and MDPs, respectively (13, 15). These subsets have been shown to exhibit distinct responses to inflammatory stimuli and differing capacities to seed tissue-resident macrophage pools (14, 16–18). To our knowledge, our study is the first to systematically characterize monocyte heterogeneity in the context of chemotherapy-induced myelopoiesis. Our scRNA-seq analysis of pre-enriched classical monocytes confirms the presence of both NeuMo-like and DCMo-like cells in steady-state BM. Moreover, our data demonstrate that chemotherapy-induced myelopoiesis leads to preferential expansion of NeuMo cells (Figure 3). The monocyte heterogeneity and skewed differentiation toward NeuMo after chemotherapy are also reflected at the chromatin level revealed by ATAC-seq (Figure 4).

It has long been reported that CTX induces immunosuppressive myeloid cells (26–28), and our previous work identified monocytes as the primary immunosuppressive population (19, 20). The current study provides direct evidence that the immunosuppressive activity of CTX-induced monocytes is confined exclusively to the NeuMo subset (Figure 6). The immunoregulatory function of chemotherapy-induced NeuMo cells, as reflected by their capacity to inhibit T cell activation and suppress proinflammatory cytokine production, is consistent with previous reports implicating NeuMo cells in the resolution of inflammation and tissue injury (14, 18, 21). Importantly, our observation of immunosuppressive monocytes emerging in the PBMCs of some lymphoma patients receiving SOC chemotherapy highlights the potential clinical relevance of this myeloid subset, whose immunomodulatory role may have been previously overlooked. Prior studies have associated the presence of chemotherapy-induced immunosuppressive myeloid cells with poor clinical outcomes in patients with breast cancer or melanoma (28, 29). However, it remains unclear whether the immunosuppressive myeloid cells identified in these patients are indeed NeuMo-like. A major obstacle to resolving this question is the lack of definitive surface markers for human NeuMo cells, which limits their identification and functional characterization. Ikeda et al. identified CD14^+^CD16^–^CXCR1^+^ monocytes in healthy human PBMCs as potential counterparts to mouse NeuMo cells (21). These cells share certain functional properties with mouse NeuMo, including immunosuppressive capacity and responsiveness to G-CSF, but they do not express canonical NeuMo gene signatures such as *MPO*, *ELANE*, *CTSG*, or *PRTN3*. In contrast, our reanalysis of publicly available scRNA-seq datasets from breast and head and neck cancer patients revealed a population of CD14^–^CD16^–^ monocytes that emerge following chemotherapy and express a NeuMo gene signature, including *MPO*, *ELANE*, and *CTSG* (Figure 7). Notably, these cells also exhibit high proliferative potential, as indicated by the expression of *MKI67*, *TOP2A*, and *STMN1*, resembling mouse NeuMo and suggesting that they are early progenitors directly derived from GMPs. The discrepancy between our findings and those of Ikeda et al. suggests that human NeuMo cells may differ between steady-state and stress-induced myelopoiesis.

NeuMo cells expanded during systemic inflammation, such as those induced by lipopolysaccharide or surgical procedures, have been shown to promote metastasis in mice (25). A recent study further reported that chemotherapy with gemcitabine or paclitaxel plus doxorubicin leads to the accumulation of monocytes in the lungs that promote metastatic progression (30). Notably, the metastasis-promoting monocytes in this study express several hallmark NeuMo signature genes, including *Prtn3* and *Elane*, suggesting that NeuMo expansion may be a common feature of chemotherapy-induced myelopoiesis. To investigate whether CTX-induced NeuMo cells exhibit similar pro-tumorigenic activity, we used an orthotopic breast cancer model (EMT6.luci) combined with adoptive transfer of monocytes (Supplemental Figure 6A). Owing to technical constraints in isolating sufficient numbers of NeuMo cells for repeated infusions, the total monocyte population (CD11b^+^Ly6C^hi^) from the BM of CTX-treated mice, enriched for NeuMo cells, was used for adoptive transfer. Control mice were implanted with EMT6.luci cells only. Infusion of CTX-induced monocytes did not influence primary tumor growth in comparison with controls (Supplemental Figure 6, B and C). As expected for the poorly metastatic EMT6 model (31, 32), control mice showed no detectable metastatic signals in distant organs at the experimental endpoint. However, mice infused with CTX-induced monocytes displayed clear metastatic signals in multiple distant sites, including the lung, liver, and intestine, as revealed by ex vivo BLI (Supplemental Figure 6D). It is plausible that NeuMo cells may facilitate the colonization of distant organs by dormant disseminated tumor cells, potentially through metastatic niche conditioning and/or suppression of antitumor T cell responses. Further investigation is warranted to elucidate the mechanisms by which NeuMo cells contribute to metastatic progression.

Despite these insights, our study has several limitations. Although our retrospective analysis revealed the emergence of de novo immunosuppressive monocytes in the PBMCs of some patients following SOC chemotherapy, the sample size was limited, and the precise molecular identity of these monocytes remains undefined. Elucidating the relationship between NeuMo abundance, patient characteristics, and clinical outcomes will require larger-scale prospective studies. It is also well established that tumor-derived factors can alter myelopoiesis (9, 33–37). Numerous studies have shown that tumor-induced myeloid-derived suppressor cells (MDSCs), which contain monocytic and granulocytic subsets, can inhibit antitumor immune responses and promote tumor progression, angiogenesis, and metastasis (38). While work from our group and others suggests that immunosuppressive myeloid cells induced by tumors are distinct from those induced by chemotherapy (20, 39), the two populations currently cannot be reliably distinguished based on phenotypic markers alone. To specifically examine chemotherapy-induced reactive myelopoiesis, we conducted experiments in tumor-free mice treated with CTX to avoid potential confounding effects from tumor-derived signals. However, it remains to be determined whether tumor-induced MDSCs contain a NeuMo-like subset, and how the presence of tumors may influence the trajectory and outcome of chemotherapy-induced myelopoiesis.

In summary, our study identifies NeuMo as a distinct subset of chemotherapy-induced monocytes with immunosuppressive activities. Their emergence in both mice and cancer patients following chemotherapy suggests a conserved, stress-induced myeloid response with the potential to impair treatment outcomes. These findings underscore the need to consider the unintended immunological consequences of chemotherapy. Therapeutic strategies targeting NeuMo cells may mitigate their immunosuppressive effects and enhance the efficacy of chemotherapy.

## Methods

### Sex as a biological variable.

Sex was not considered as a biological variable in this study. Both male and female mice were used for analysis except for experiments using the EMT6 breast cancer model. For studies using human samples, cryopreserved, deidentified patient blood samples were analyzed based on availability, without regard to sex. The potential influence of sex differences on the emergence of chemotherapy-induced immunosuppressive myeloid cells remains unknown.

### Antibodies and reagents.

Single-cell suspensions were stained for flow cytometry analysis using fluorochrome-conjugated antibodies specific to murine CD4 (GK1.5) (BioLegend, 100407), CD8 (53-6.7) (BioLegend, 100712), CD25 (3C7) (BioLegend, 101908), CD11b (M1/70) (BioLegend, 101215), Ly6C (HK1.4) (BioLegend, 128025), CXCR4 (L276F12) (BioLegend, 146511), and CX3CR1 (SA011F11) (BioLegend, 149005), and antibodies specific to human CD4 (OKT4) (BioLegend, 317407), CD8a (HIT8a) (BioLegend, 300912), CD11b (ICRF44) (BioLegend, 301317), CD14 (M5E2) (BioLegend, 301820), CD33 (WM53) (BioLegend, 303404), and HLA-DR (L243) (BioLegend, 307616). Cyclophosphamide (CTX) was purchased from Tokyo Chemical Industry (C2236).

### Patient samples.

Peripheral blood samples were collected from individuals with B cell lymphoma scheduled to receive either R-CHOP (rituximab, cyclophosphamide, doxorubicin, vincristine, and prednisone) or R-EPOCH (rituximab, etoposide, cyclophosphamide, doxorubicin, vincristine, and prednisone) chemotherapy. Patients were treated every 21 days and received a total of 6 cycles to complete their initial induction chemotherapy. Blood samples were collected before (within 60 days) the first chemotherapy and after the third or fourth cycle. Informed consent documents were provided to patients to obtain their permission to use their blood samples for research purposes. The blood was collected during their routine lab draw on the day of treatment. The specimens were deidentified and preserved in the Georgia Cancer Center Biorepository. Available patient information is summarized in Supplemental Table 1.

### Mice.

BALB/c mice 4–8 weeks of age were purchased from Charles River Laboratories. All mice were housed under specific pathogen–free conditions by Laboratory Animal Services of Augusta University.

### Sample preparation for flow cytometry analysis and cell sorting.

For mouse bone marrow (BM) preparation, tibia and femur bones were collected from euthanized mice. BM was collected by flushing of the bones with PBS. Red blood cells were removed using ammonium-chloride-potassium (ACK) lysing buffer to obtain BM single-cell suspensions. For human samples, white blood cells (PBMCs) were isolated from cryopreserved blood by density gradient centrifugation using Ficoll-Paque PLUS (Sigma-Aldrich, GE17-1440-03). For flow cytometry analysis, single-cell suspensions from mouse BM or human PBMCs were stained with fluorochrome-conjugated antibodies for 15 minutes at room temperature in the dark. All flow cytometry analysis data were acquired on an Attune NxT Flow Cytometer (Invitrogen) and analyzed using FlowJo software (Tree Star). To isolate different subsets of myeloid cells, cells stained with the specified antibodies were subjected to cell sorting on a Bigfoot Spectral Cell Sorter (Invitrogen).

### In vitro T cell suppression assay.

For mouse samples, spleens collected from normal BALB/c mice were ground thoroughly in PBS, and the suspensions were passed through a 70 μm nylon mesh to remove debris. After lysing of red blood cells with ACK buffer, spleen cells were labeled with 0.1 μM CellTrace violet dye (Invitrogen,C34557) and seeded in triplicate into a round-bottom 96-well plate with 1 × 10^5^ cells per well in 200 μL medium. T cells (both CD4^+^ and CD8^+^) within spleen cells were stimulated with 1 μg/mL of anti-CD3 (BioLegend, clone 145-2C11, 100301) and 5 μg/mL of anti-CD28 (BioLegend, clone 37.51, 102102). 1 × 10^5^ myeloid cells sorted by fluorescence-activated cell sorting (FACS) were added to the specified wells. Cells were harvested 3 days after culture and stained with antibodies against CD4, CD8, and CD25 for flow cytometry analysis. Cell culture supernatants were collected and used to quantify cytokines. The human T cell suppression assay was set up summarily as described above. T cells isolated from a healthy donor were labeled with CellTrace violet dye and used as responder cells. T cells were stimulated with Human T-Activator CD3/CD28 Dynabeads (Invitrogen, 11131D) in the presence of monocytes (HLA-DR^–^CD11b^+^CD14^+^CD33^+^) sorted from patients’ PBMCs. T cells were harvested 3 days later to evaluate cell division and activation status in CD4^+^ and CD8^+^ T cells by FACS.

### Quantification of cytokines in cell culture supernatants.

Detection of selected cytokines (IL-2, IL-4, IL-6, IL-9, IFN-γ, TNF-α) in cell culture was performed using LEGENDplex Mouse TH Panel V03 (BioLegend, 741044) according to the manufacturer’s instructions. In brief, standards and samples were plated with capture beads and incubated overnight at 4°C on a plate shaker (0.02 × *g*). After washing of the plate, detection antibodies were added to each well, and the plate was incubated on a shaker (0.06 × *g*) for 1 hour at room temperature. Finally, without washing, streptavidin-phycoerythrin (SA-PE) was added and incubated for 30 minutes. Samples were acquired on a Novocyte Quanteon flow cytometer (Agilent Technologies). Standard curves and protein concentration were calculated using the R package DrLumi installed on R 3.6.1. The limit of detection was calculated as an average of background samples plus 3 × SD.

### Single-cell RNA sequencing.

For scRNA-seq of whole BM cells, BM was collected from cyclophosphamide-treated mice on day 2 (D2 CTX) and day 7 (D7 CTX). BM from untreated mice was used as the control (naive). Live cells were enriched using the EasySep Dead Cell Removal kit (StemCell Technologies, 17899). Equal numbers of cells from 3 individual mice under each condition were mixed, and the pooled samples were used for downstream sequencing. For scRNA-seq of pre-enriched monocytes, BM cells were stained with anti-CD11b and anti-Ly6C antibodies and sorted for CD11b^+^Ly6C^hi^ monocytes by a Bigfoot Spectral Cell Sorter. The purity of sorted monocytes was greater than 98%. Freshly isolated whole BM cells or monocytes (CD11b^+^Ly6C^hi^) were processed for scRNA-seq libraries using the Chromium Controller (10x Genomics). scRNA-seq libraries were generated using 10x 3′ single-cell mRNAseq V3 reagents (10x Genomics, 1000075) and sequenced using an Illumina NovaSeq 6000 sequencer.

### scRNA-seq data analysis.

The raw reads were produced using the 10x Genomics Cell Ranger v3.1 pipeline. Samples were pooled using the cellranger aggr pipeline. The cellranger pipeline outputs containing gene-by-cell expression data were processed using the R package Seurat v5.0. Graphs were generated using plotting functions in the Seurat (https://satijalab.org/seurat/) and scCustomize v3.01 (https://samuel-marsh.github.io/scCustomize/) packages. Data quality control measures were implemented using Seurat, and only cells with at least 200 genes and less than 20% of mitochondria gene content were retained for further analysis. Normalization, scaling, dimension reduction, integration, and clustering were carried out using Seurat. The top differentially expressed marker genes for each cluster were identified by built-in Seurat functions. Differential gene expression analysis between NeuMo and other monocyte clusters was performed using the MAST test implemented in Seurat, and the Bonferroni method was used for multiple-comparison correction. The myeloid precursors and mature myeloid cells in BM were identified by gene signatures published by Kwok et al. (40). NeuMo and DCMo subsets in the sorted monocyte population were identified by gene signatures published by Goodridge’s group (13, 16). The NeuMo gene signature score was calculated using AUCell v1.2 (https://github.com/aertslab/AUCell).

### RNA velocity analysis.

RNA velocity analysis was performed using the Python package scVelo v0.2.5 (https://scvelo.readthedocs.io/en/stable/), which uses a modified dynamical model to relate the dynamics of unspliced pre-mRNA and spliced mature RNA. The BAM files generated by the cellranger pipeline were pre-processed using the velocyto command-line interface (CLI) tool (https://velocyto.org/) with default parameters specific for 10x Genomics data. RNA velocity was estimated using default parameters in scVelo. Velocity graphs were generated using functions in scVelo and Scanpy based on the UMAP embedding generated by Seurat analysis. scVelo uses RNA velocity to infer latent time, which can be interpreted as a type of pseudotime but is based on transcriptional dynamics, not just transcriptomic similarity.

### ATAC sequencing analysis.

Nuclei were prepared from freshly isolated monocytes (CD11b^+^Ly6C^hi^) by FACS and used for the ATAC-seq library construction as described previously (41). Briefly, nuclei were incubated with 2.5 mL Nextera Tn5 transposase (Illumina, 20034197) in 50 μL 1× transposition reaction at 37°C for 1 hour. The transposition reaction mixtures were purified with the DNA Clean and Concentrator Kit (Zymo Research, D4014), and amplified for 11–13 cycles using NEBNext HighFidelity 2× Master Mix (New England Biolabs, M0541L) and Nextera Index primers (Illumina, FC-121-1011). The libraries were size-selected and sequenced on a HiSeq 4000 instrument in a paired-end 150-cycle run. Raw reads in FASTQ format were first examined with FastQC v0.11.8 for quality control. Adapter and quality trimming of the raw sequencing reads was performed using Trim Galore! v0.6.3. Trimmed reads were aligned to the mouse reference genome mm10 using Bowtie 2 v2.3.4.1. Reads aligned to the mitochondrial genome were filtered using SAMtools v1.8. PCR duplicates were removed using MarkDuplicates from Picard v2.18.2. The aligned reads overlapping with ENCODE blacklist regions (version 2) were removed using SAMtools. ATAC-seq peak calling was performed using MACS2 v2.1.1.20160309. Differential ATAC-seq peaks between the naive and CTX groups (*n* = 3 per group) were identified using DiffBind v2.10.0. The nearest genes with a ±5,000 bp flanking distance of differential peaks were annotated using ChIPseeker v1.4. BigWig files were generated by the deepTools bamCoverage function and visualized in Integrative Genomics Viewer v2.8.3 (https://igv.org/). The above analyses were perform using tools available on the open source galaxy server (https://usegalaxy.org/). The above analyses were perform using tools available on the open source galaxy server (https://usegalaxy.org/). The bigWig track plot for the individual locus was generated using karyoploteR v1.16 in R v4.4. Heatmaps were generated using ComplexHeatmap v2.22. Pathway enrichment analysis and gene set enrichment analysis (GSEA) were performed using clusterProfiler v4.14.4. Both packages are available through Bioconductor (https://bioconductor.org/).

### Analysis of publicly available human PBMC samples.

Two publicly available scRNA-seq datasets of paired PBMC samples pre- and post-chemotherapy were downloaded from GEO (GSE260953 and GSE263995). The count matrix files and associated metadata were used to construct the Seurat objects, which were then analyzed in the same manner as described above. Only cells with at least 300 genes and less than 20% of mitochondrial gene content were retained. Doublets were identified by the R package DoubletFinder v2.0.4 (https://github.com/chris-mcginnis-ucsf/DoubletFinder), and only singlets were retained for further analysis. The PBMC subtypes were annotated using Azimuth v0.5.0 packages based on built-in human PBMC reference datasets. Monocytes were extracted from the whole PBMC datasets and reintegrated using Seurat reciprocal PCA (RPCA) integration, a more conservative integration approach. Differential gene expression analysis and visualization plotting were carried out as described above. Pseudo-bulk gene expression analysis was performed using Seurat, and a volcano plot was generated with the EnhancedVolcano v1.24.0 (https://bioconductor.org/) package in R v4.4.

### Statistics.

Data were analyzed using Prism 7.0 (GraphPad Software Inc.). For comparison between 2 groups, the statistical significance of the results was determined using unpaired 2-tailed Student’s *t* test. For pairwise comparisons, 2-sided Wilcoxon’s rank-sum tests were performed. One-way ANOVA was used to determine statistical differences among 3 or more groups. For multiple-testing correction, the false discovery rate (FDR) method was used. The adjusted *P* values described in this article refer to the FDR, unless otherwise stated. A *P* value less than 0.05 was considered significant.

### Study approval.

Experiments using human materials were approved by the Institutional Biosafety Committee of Augusta University, and written informed consent was obtained prior to blood draws. Murine experiments were performed in accordance with the NIH *Guide for the Care and Use of Laboratory Animals* (National Academies Press, 2011) and were approved by the Institutional Animal Care and Use Committee of Augusta University.

### Data availability.

Sequencing data were deposited in the Gene Expression Omnibus (GEO) under accession number GSE314232. Open-source tools were used to analyze the data, and custom scripts for processing raw data and generating figures will be made available upon request.

## Author contributions

HS designed the study, performed data analysis, interpreted results, and wrote the manuscript. ZD, ODO, XW, GIZ, YY, MYG, CB, LP, and RP conducted experiments and analyzed data. CCH and LJB advised on study design and edited the manuscript. GZ designed the study, conducted experiments, interpreted results, and wrote the manuscript.

## Conflict of interest

The authors have declared that no conflict of interest exists.

## Funding support

This work is the result of NIH funding, in whole or in part, and is subject to the NIH Public Access Policy. Through acceptance of this federal funding, the NIH has been given a right to make the work publicly available in PubMed Central.

- NIH CA215523 (to GZ).
- NIH CA238514 (to GZ).
- NIH CA264983 (to GZ and HS).
- NIH P01-HL136275 (to CCH).
- NIH P01-HL152958 (to CCH).
- Paceline (to GZ).
- Evans County CARES Inc. (to HS).

## Supplementary Material

Supplemental data

Supporting data values

## Version 1 04/07/2026

In-Press Preview

## Version 2 05/22/2026

Electronic publication
