# Supplementary material for: Chemotherapy-induced reactive myelopoiesis promotes expansion of immunosuppressive neutrophil-like monocytes in mice and humans
Source: JCI Insight. 2026 Mar 31;11(10):e198360. doi: 10.1172/jci.insight.198360 (PMC13232731; doi:10.1172/jci.insight.198360)
Supplement: Supplemental data [file jciinsight-11-198360-s029.pdf]

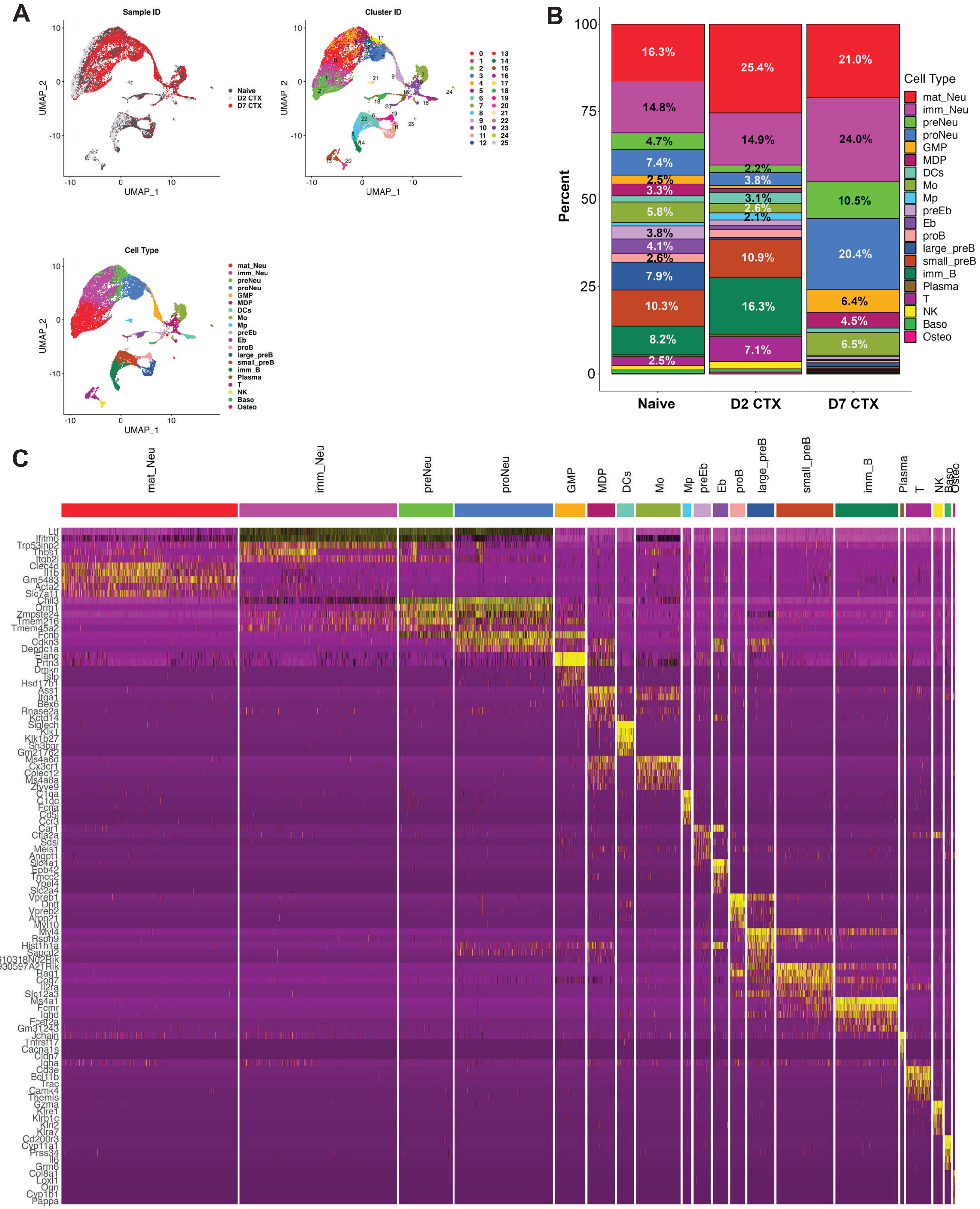
A
Sample ID
UMAP_2
UMAP_1
Naive
D2 CTX
D7 CTX
Cluster ID
0 1 2 3 4 5 6 7 8 9 10 11 12 13 14 15 16 17 18 19 20 21 22 23 24 25
Cell Type
mat_Neu
imm_Neu
preNeu
proNeu
GMP
MDP
DCs
Mo
Mp
preEb
Eb
proB
large_preB
small_preB
imm_B
Plasma
T
NK
Baso
Osteo
B
Percent
100
75
50
25
0
16.3%
14.8%
4.7%
7.4%
2.5%
3.3%
5.8%
3.8%
4.1%
2.6%
7.9%
10.3%
8.2%
2.5%
25.4%
14.9%
2.2%
3.8%
3.1%
2.6%
2.1%
10.9%
16.3%
7.1%
21.0%
24.0%
10.5%
20.4%
6.4%
4.5%
6.5%
Naive
D2 CTX
D7 CTX
Cell Type
mat_Neu
imm_Neu
preNeu
proNeu
GMP
MDP
DCs
Mo
Mp
preEb
Eb
proB
large_preB
small_preB
imm_B
Plasma
T
NK
Baso
Osteo
C
mat_Neu
imm_Neu
preNeu
proNeu
GMP
MDP
DCs
Mo
Mp
preEb
Eb
proB
large_preB
small_preB
imm_B
Plasma
T
NK
Baso
Osteo
Ltf
Ifitm6
Trp53inp2
Thbs1
Itgb2l
Clec4d
Il1b
Gm5483
Acta2
Slc7a11
Chil3
Orm1
Zmpste24
Tmem216
Tmem45a2
Fcnb
Cdkn3
Depdc1a
Elane
Prtn3
Dmkn
Tslp
Hsd17b1
Ass1
Itga1
Bex6
Rnase2a
Kctd14
Siglech
Klk1
Klk1b27
Sh3bgr
Gm21762
Ms4a6d
Cx3cr1
Colec12
Ms4a8a
Zfyve9
C1qa
C1qc
Fcna
Cd5l
Ccr3
Car1
Ctla2a
Sdsl
Meis1
Angpt1
Slc4a1
Epb42
Tmcc2
Ypel4
Slc2a4
Vpreb1
Dntt
Vpreb2
Arpp21
Myl10
Myl4
Rsph9
Hist1h1a
Sapcd2
2610318N02Rik
4930597A21Rik
Rag1
Cpq7
Il2ra
Slc12a3
Ms4a1
Fcmr
Ighd
Fcer2a
Gm31243
Jchain
Tnfrsf17
Cacna1s
Cldn7
Igha
Cd3e
Bcl11b
Trac
Camk4
Themis
Gzma
Klre1
Klrb1c
Klri2
Klra7
Cd200r3
Cyp11a1
Prss34
Il6
Grm6
Col8a1
Loxl1
Ogn
Cyp1b1
Pappa

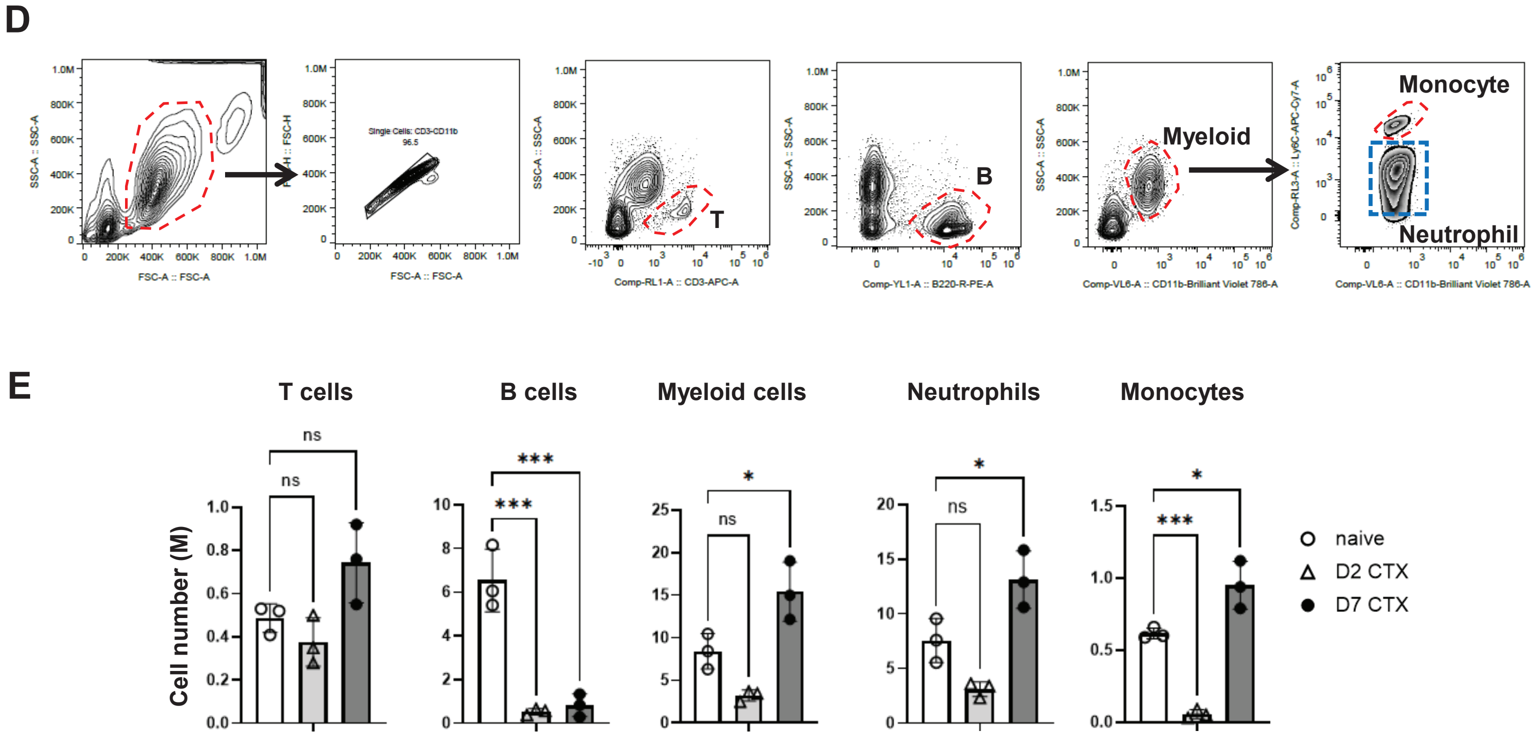

Supplemental Figure 1. A, UMAP plots showing annotated cell populations based on gene expression profiles in BM samples from naïve, Day 2, and Day 7 CTX-treated mice. B, Bar graph summarizing the proportions of each identified cell type across the three conditions. C, Heatmap of top 10 genes in each cluster identified in BM scRNA-seq analysis. The columns represent cells, and the rows present genes. Cells are grouped by clusters, and the top 10 most significant markers are shown. D. Representative dot plots illustrate the gating strategy used to determine the frequencies of the indicated cell subsets. The tibia and femur bones from both legs of naïve and CTX-treated mice were collected for analysis. BM cells were enumerated and stained with antibodies against CD3, B220, CD11b, Ly6G and Ly6C for flow cytometry analysis. E. The calculated absolute numbers of each specified BM cell subset are summarized in the bar graph (mean ± SEM) with 3-4 mice each group. Data shown are representative of two independent experiments with similar results. Statistical analysis was performed using one-way ANOVA Tukey's multiple comparison test. *, P<0.05; **, P<0.01; ***, P<0.001; ns, not significant.

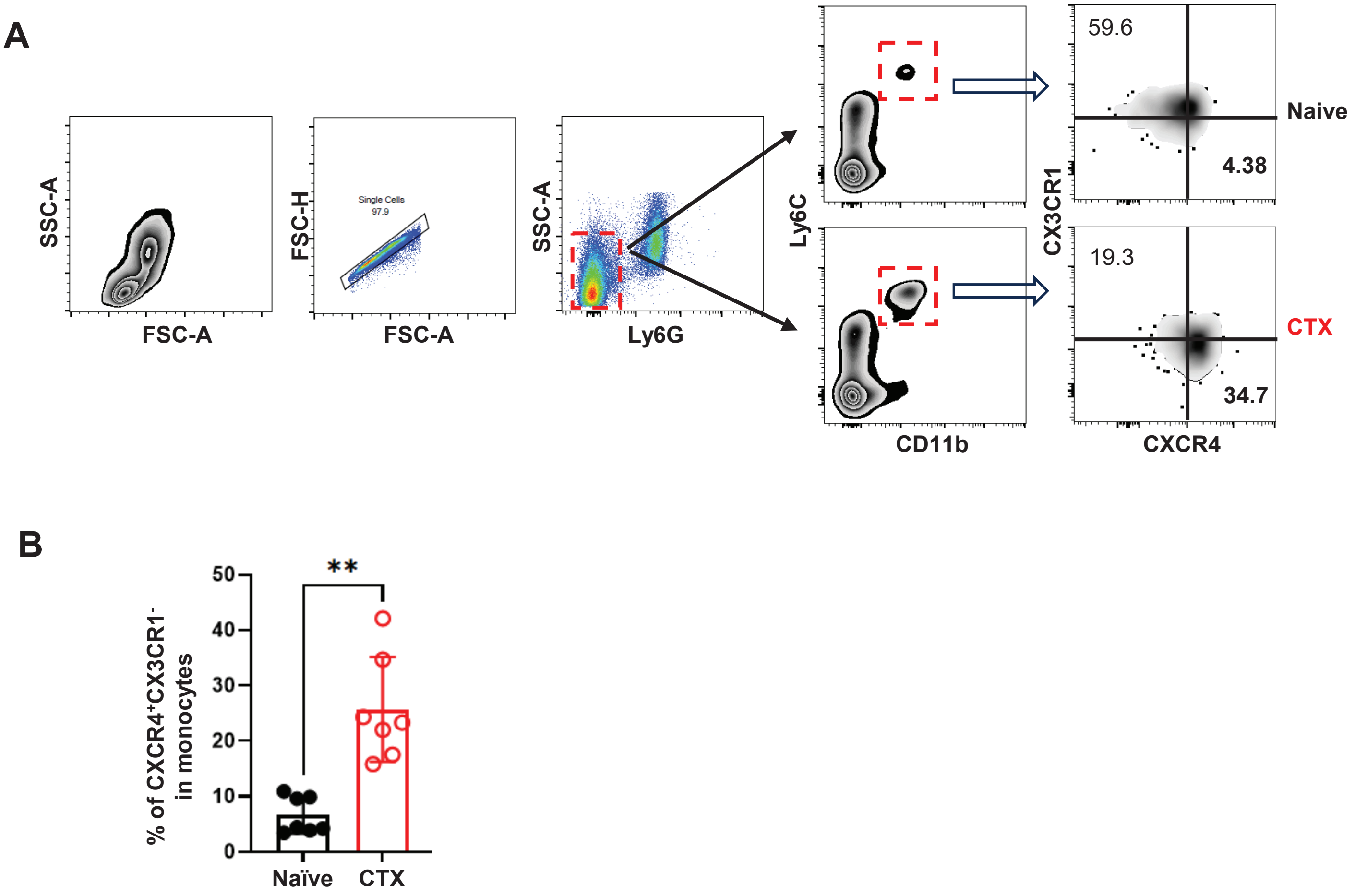

Supplemental Figure 2. Increased frequency of CXCR4$^+$CX3CR1$^-$ monocytes in the peripheral blood of CTX-treated mice. Blood samples from naïve and CTX-treated mice (day 7 post-treatment) were processed to remove red blood cells and stained with antibodies against CD11b, Ly6G, Ly6C, CXCR4, and CX3CR1, followed by flow cytometric analysis. A. Representative dot plots show the gating strategy and CXCR4/CX3CR1 expression profiles within the CD11b$^+$Ly6C^{hi} monocyte population. B. The percentage of CXCR4$^+$CX3CR1$^-$ NeuMo-like monocytes among total monocytes is summarized in the bar graph (mean ± SEM) with 7 mice per group combined from two independent experiments. Statistical analysis was performed using 2-tailed unpaired t test with Welch's correction. **, P<0.01.

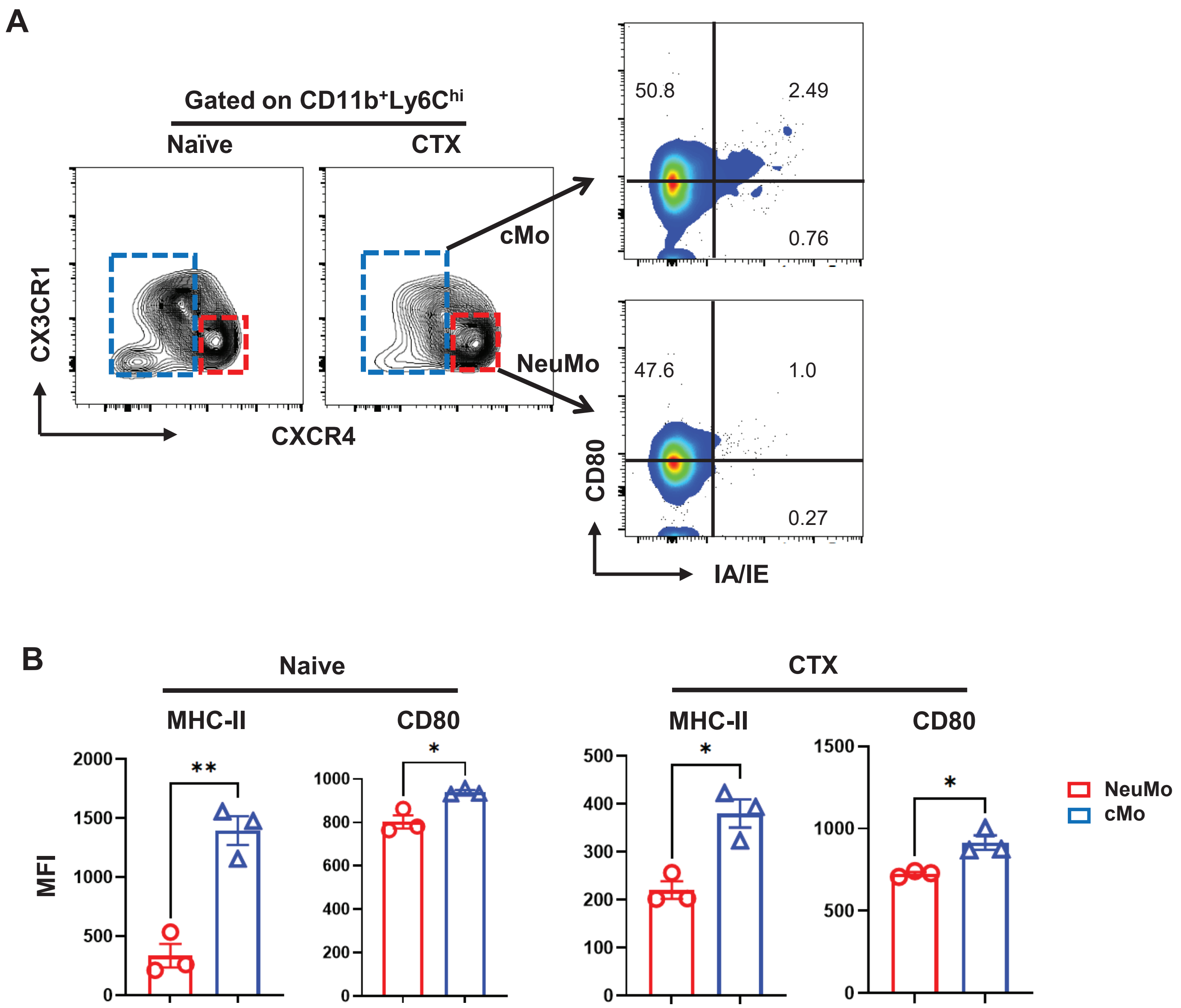

Supplemental Figure 3. NeuMo-like cells express lower levels of MHC-II and CD80 compared to classical monocytes. BM samples from naïve and CTX-treated mice (day 7 post-treatment) were stained with antibodies against CD11b, Ly6G, Ly6C, CXCR4, CX3CR1, CD80 and IA/IE followed by flow cytometric analysis. A. Representative dot plots show the gating strategy and MHC-II/CD80 expression profiles within the $CD11b^{+}Ly6G^{-}Ly6C^{hi}$ monocyte population. B. The mean fluorescence intensities (MFI) of MHC-II and CD80 of NeuMo-like and cMo subsets monocytes are summarized in the bar graph (mean ± SEM) with 3 mice per group. Data shown are representative of two independent experiments with similar results. Statistical analysis was performed using 2-tailed unpaired t test with Welch’s correction. *, P<0.05; **, P<0.01.

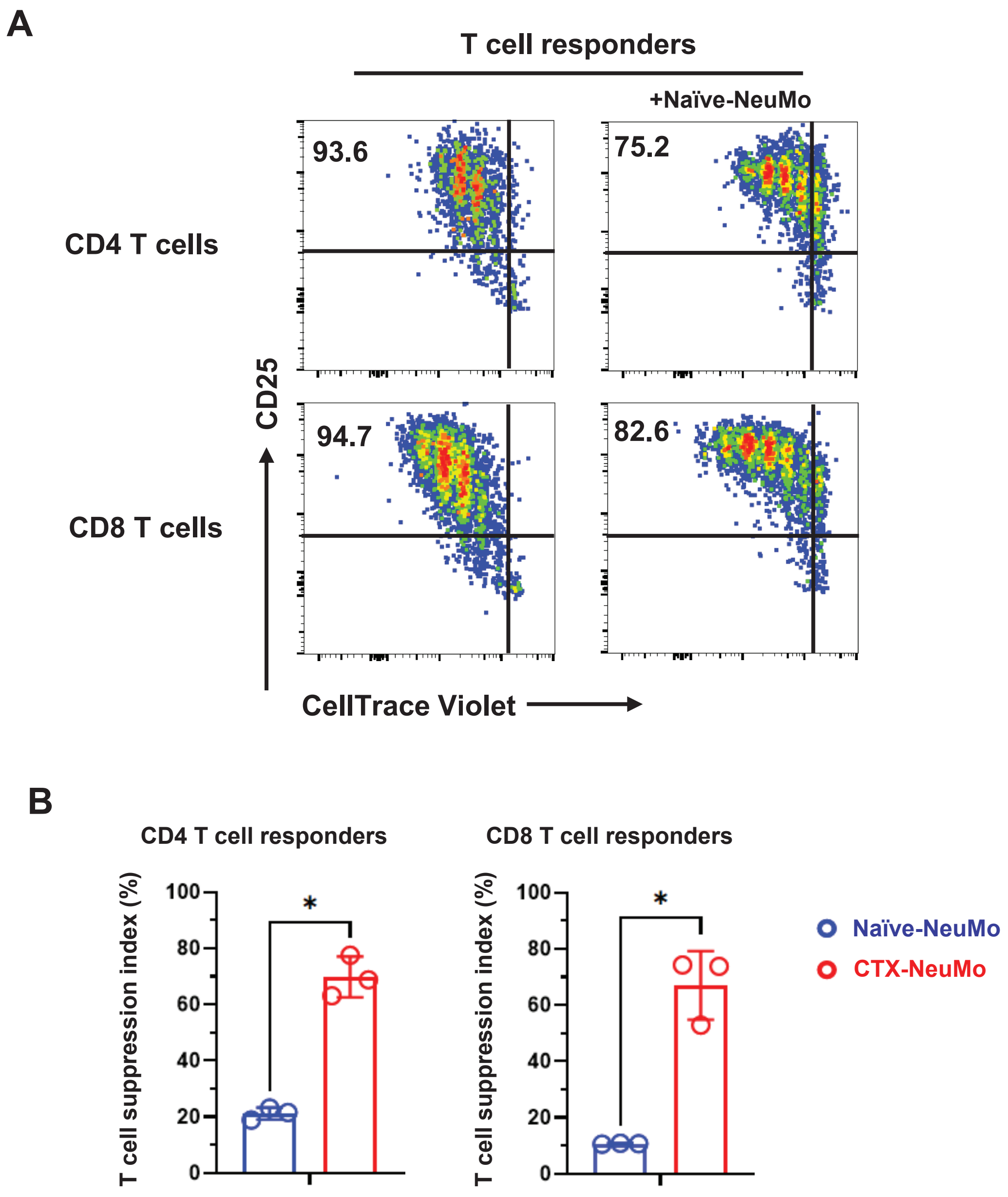

Supplemental Figure 4. NeuMo-like cells from the BM of naïve mice have weak immunosuppressive activity. NeuMo-like cells (CD11b^{+}Ly6C^{hi}CXCR4^{hi}CX3CR1^{lo}) were FACS-sorted from the BM of naïve BALB/C mice. The sorted cells were used for in vitro T cell suppression assay following similar procedures described in Fig. 6. 3 days after culture, cells were harvested for analysis. A. Representative dot plots showing cell division and activation status of the responder T cells. Cells were stained with antibodies against CD4, CD8 and CD25. Representative dot plots are gated on CD4+ (top panel) and CD8+ (bottom panel) T cells to show cell division status (violet dye dilution) and CD25 expression level. Numbers in dot plots represent percent of fully activated T cells (divided CD25^{hi}) under the specified culture condition. B. Comparison of T cell suppression potency of BM NeuMo-like cells from naïve mice and CTX-treated mice. The ability of sorted myeloid cells to suppress T cell activation is measured by T cell suppression index calculated using the formula: (% of divided CD25^{hi} T cells in T cell only culture - % of divided CD25^{hi} T cells in co-culture) / (% of divided CD25^{hi} T cells in T cell only culture). Data for CTX-NeuMo are calculated from the data shown in Fig. 6C. The results are summarized in bar graphs shown as mean±SEM of triplicate samples. Statistical analysis was performed using 2-tailed unpaired t test with Welch's correction. *, $P<0.05$.

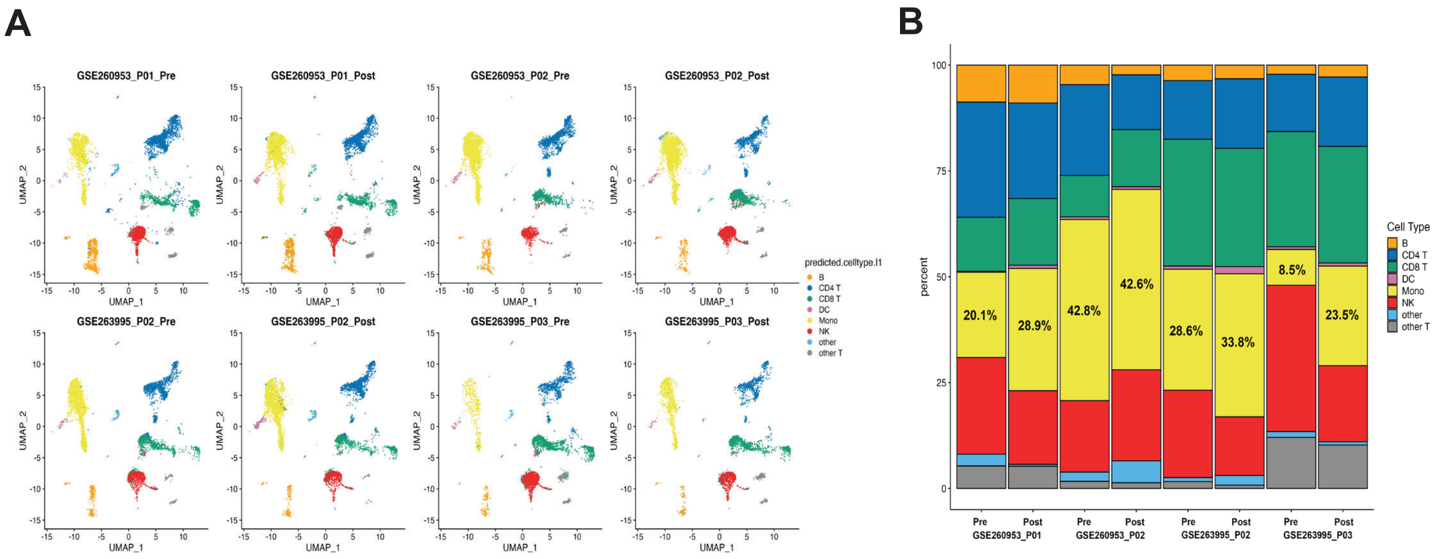

Supplemental Figure 5. Reanalysis of publicly available scRNA-seq data in cancer patients receiving chemotherapy. scRNA-seq datasets were downloaded from GEO and analyzed using Seurat. A. UMAP plots showing annotated cell populations based on gene expression profiles in paired pre-treatment and post-treatment samples from 4 cancer patients, including two patients with tongue squamous cell carcinoma, treated with docetaxel, cisplatin, and 5-fluorouracil (GSE260953_P01 and GSE260953_P02) and two TNBC patients treated with cyclophosphamide/doxorubicin (GSE263995_P02 and GSE263995_P03). scRNA-seq data were reference-mapped using the Azimuth PBMC dataset to annotate cell types. B. Bar plot representing the proportions of major cell types in pre- and post-chemotherapy samples.

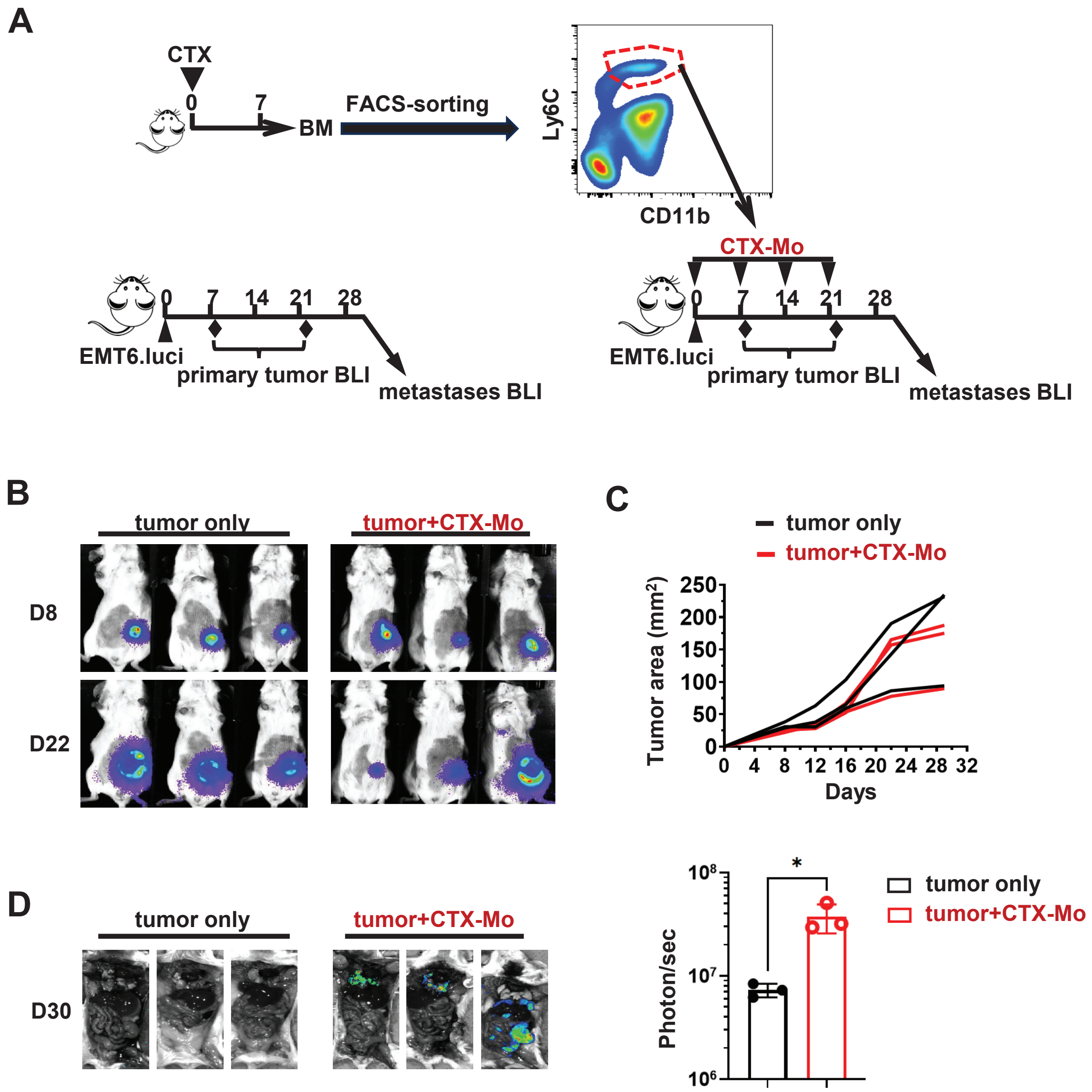

Supplemental Figure 6. CTX-induced monocytes exhibit pro-metastatic capacity in an orthotopic EMT6 breast cancer model. A. The experimental procedures and timelines are depicted in the schema. Briefly, naïve mice received intraperitoneal injection of CTX at the dose of 150 mg/kg. 7 days later, BM cells were collected from these mice. Monocytes (CTX-Mo) were FACS-sorted to high purity (>98%) and infused to recipient mice via tail vein injection. Mice were implanted with EMT6.luci cells in the mammary fat pads immediately after the first infusion of sorted myeloid cells. One cohort of mice received tumor inoculation only were included as control. Infusions of FACS-sorted CTX-induced monocytes were repeated on day 7, day 14 and day 21. B. The burdens of the primary tumors were evaluated by BLI at the specified time points. C. Tumor growth curves of the primary tumors. Tumor areas (length x width) are plotted against time with 3 mice per group. D. Detection of metastases in distant organs by BLI on day 30. All mice were euthanized on day 30. The primary tumors were excised, and the internal organs of each mouse were subjected to BLI. Results of luciferase signal intensity (photon/sec) in distant organs quantified as mean$\pm$SEM are summarized in the bar graph at right. Statistical analysis was performed using 2-tailed unpaired t test with Welch's correction. *, $P<0.05$.

Supplemental Table I. Patient Characteristics

Sample ID	Age (yrs)	Sex	Diagnosis	Stage	Subtype/Features	NCCN-IPI Score	Treatment	Best Response	Outcome/Status (at time of manuscript preparation)
15801	73	F	DLBCL	IIA	non-GCB	2 (low-int)	R-CHOP ×6	CR	Alive, in remission
143735	80	F	DLBCL	IVB	GCB, double-hit	6 (high)	R-CHOP ×6	CR	Died (accidental fall, 6 mo post-CR)
15874	19	M	ALCL (ALK+)	IVB	ALK+		CHOEP ×6	CR	Relapsed at 13 mo; lost to follow-up
16060	78	F	DLBCL	IVA	GCB	6 (high)	R-CHOP ×6	CR	Alive, in remission
16093	78	F	DLBCL	IA	non-GCB	5 (high-int)	R-CHOP ×6 + IFRT	Brief CR	Relapsed in 4 mo; deceased
1520242	32	M	DLBCL	IIIB	GCB	1 (low)	R-CHOP ×6	CR	Alive, in remission
16679	21	F	NLPHL	IIIA			R-CHOP ×6	CR	Alive, in remission
16772	70	F	DLBCL	IVB	non-GCB	5 (high-int)	R-CHOP ×6 (reduced dose)	CR	Relapsed in 6 mo; deceased

DLBCL: diffuse large B cell lymphoma

ALCL: anaplastic large cell lymphoma

NLPHL: nodular lymphocyte-predominant B-cell lymphoma

GCB: germinal center B-cell

R-CHOP: Rituximab, Cyclophosphamide, Hydroxydaunorubicin, Oncovin, and Prednisone

CHOEP: Cyclophosphamide, Hydroxydaunorubicin, Oncovin, Etoposide, and Prednisone

IFRT: Involved-Field Radiotherapy
